# Real-time monitoring of PARP1-dependent PARylation by ATR-FTIR spectroscopy

**DOI:** 10.1038/s41467-020-15858-w

**Published:** 2020-05-01

**Authors:** Annika Krüger, Alexander Bürkle, Karin Hauser, Aswin Mangerich

**Affiliations:** 1 Department of Biology, University of Konstanz, Konstanz, 78464 Germany; 2 Department of Chemistry, University of Konstanz, Konstanz, 78464 Germany

**Keywords:** Enzyme mechanisms, Optical spectroscopy, Molecular biophysics, DNA damage response, PolyADP-ribosylation

## Abstract

Poly-ADP-ribosylation (PARylation) is a fully reversible post-translational modification with key roles in cellular physiology. Due to the multi-domain structure of poly(ADP-ribose) polymerase-1 (PARP1) and the highly dynamic nature of the PARylation reaction, studies on the biochemical mechanism and structural dynamics remain challenging. Here, we report label-free, time-resolved monitoring of PARP1-dependent PARylation using ATR-FTIR spectroscopy. This includes PARP1 activation by binding to DNA strand break models, NAD^+^ substrate binding, PAR formation, and dissociation of automodified PARP1 from DNA. Analyses of PARP1 activation at different DNA models demonstrate a strong positive correlation of PARylation and PARP1 dissociation, with the strongest effects observed for DNA nicks and 3’ phosphorylated ends. Moreover, by examining dynamic structural changes of PARP1, we reveal changes in the secondary structure of PARP1 induced by NAD^+^ and PARP inhibitor binding. In summary, this approach enables holistic and dynamic insights into PARP1-dependent PARylation with molecular and temporal resolution.

## Abstract (web summary)

The mechanism of PARP1-dependent poly-ADP-ribosylation in response to DNA damage is still under debate. Here, the authors use ATR-FTIR spectroscopy to provide time-resolved insights into the molecular details of this process under near physiological conditions.

## Introduction

Fast and efficient cellular DNA repair is essential to ensure genomic integrity over time. One of the first signalling events upon DNA damage is the recruitment of poly(ADP-ribose) polymerase-1 (PARP1) to sites of DNA damage, in particular DNA strand breaks. In this very fast process, PARP1 association rates of 10^9^ M^−1^ s^−1^ have been reported^1^. The catalytic activation of PARP1 and resulting formation of poly(ADP-ribose) (PAR) from NAD^+^ contributes to the repair of DNA lesions, stabilisation of replication forks and chromatin remodelling^2^. PAR molecules are formed by attachment of ADP-ribose units via unique ribose–ribose linkages resulting in linear and branched PAR chains, which are heterogeneous in size and branching frequencies comprising up to 200 ADP-ribose units. Initiation of PAR synthesis occurs at several amino acids in target proteins, including serine, glutamate, aspartate, lysine and tyrosine^3,4^. So far, hundreds of proteins have been identified as targets of covalent PARylation, with PARP1 being the prime target itself^5–7^. It is generally assumed that automodification of PARP1 results in steric and electrostatic repulsion and thereby in the release of PARP1 from DNA, giving access to other repair factors^8–11^.

PARP1, together with PARP2 and PARP3, belongs to the group of DNA-dependent PARPs^12^. PARP1 is activated at several different DNA structures, including single- and double-strand breaks, hairpins, cruciforms and stably unpaired regions^8,13–17^. Neither sequence specificity nor clear preferences for any DNA strand break structure have been observed so far. DNA binding is mediated via the N-terminal DNA-binding domain of PARP1 consisting of two DNA-binding zinc-fingers (ZnF1 and ZnF2) (Supplementary Fig. 1a). A third zinc finger (ZnF3) is located adjacent of ZnF2, and is involved in the functional regulation of PARP1 activity. The structural mechanism behind DNA binding has been solved by crystal and NMR structures of the ZnFs in complex with different types of DNA strand breaks^13,18–21^. Even though ZnF1, ZnF2 and ZnF3 contact DNA directly, only ZnF1 and ZnF3 are essential for PARP1 activation^13,14,22^. Robust activation of PARP1 furthermore relies on the conserved Trp–Gly–Arg (WGR) domain and the catalytic domain, consisting of the autoinhibitory helical domain (HD) and the ADP-ribosyl transferase (ART) domain (Supplementary Fig. 1a). The BRCA1 C-terminal (BRCT) domain, which is a major site of automodification and which mediates protein–protein interactions, is not essential for PARP1 activity in vitro^23–26^. Under non-activated conditions, the HD, which consists of six α-helices that form a hydrophobic core, blocks binding of NAD^+^ to the active site^27^. Upon DNA binding, PARP1 undergoes a multi-domain allosteric switch, resulting in structural distortions of the HD, and thereby the catalytic centre of the ART domain becomes accessible for NAD^+^^14,22,26–28^. While earlier data suggested a dimeric action of PARP1 and thereby a modification of PARP1 molecules in *trans*^29,30^, recent data supported the notion of a monomeric action and automodification of PARP1 in *cis*^11,14,22,31,32^. The donor site, where NAD^+^ binds, is highly conserved among PARPs. It includes a nicotinamide-binding pocket, a phosphate binding site and an adenine–ribose-binding site. The nicotinamide-binding pocket is also the site, where most pharmacological PARP inhibitors bind (Supplementary Fig. 1a). Recently, the first crystal structure of the human catalytic PARP1 domain in complex with a NAD^+^ analogue has been solved^27^.

Even though PARP1 was discovered more than five decades ago^12^, the dynamics of the PARylation reaction and consequences on PARP1 structure are still incompletely understood. While DNA binding and subsequent PARP1 activation have been studied extensively^14,22,26–28^, data on changes in PARP1 structure upon NAD^+^ binding and PARylation are largely missing. Moreover, the interplay of DNA strand break recognition by PARP1 and its dissociation upon PARylation have not been explored in detail. Since it is difficult to study large and flexible proteins using conventional methods, innovative methodologies are needed to overcome these limitations. ATR-FTIR spectroscopy is a very sensitive method suitable to study large and flexible proteins under near-physiological conditions^33,34^. Performing reaction-induced difference spectroscopy enables monitoring of small structural changes of proteins upon molecular interactions or enzymatic reactions in real time. The specific immobilisation of molecules of interest at the ATR-crystal surface enhances the local surface concentration and thus improves the signal-to-noise ratio even at low analyte concentrations, thus providing an excellent platform to study the molecular mechanisms of PARylation.

**Fig. 1:**
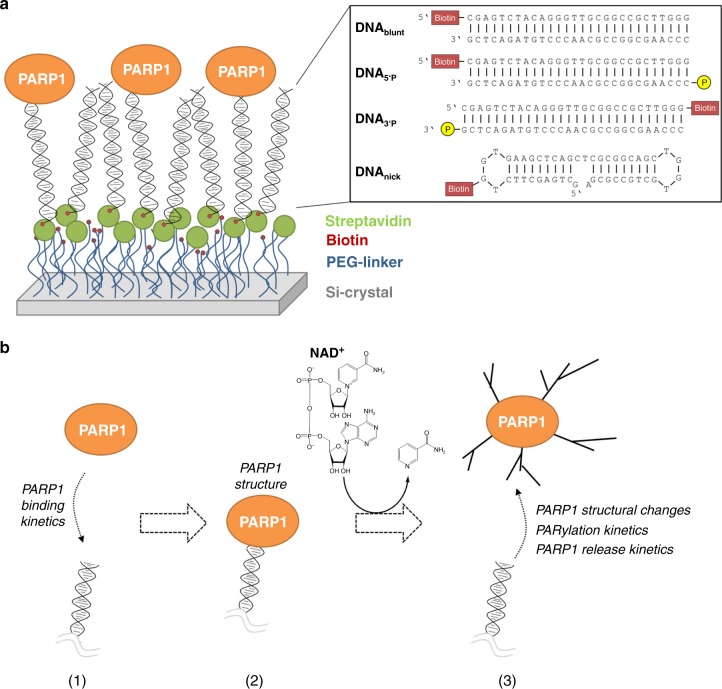
Experimental setup. **a** Schematic representation of the ATR-FTIR spectroscopic setup. The silicon crystal surface was modified with PEG linkers to prevent unspecific protein adsorption. Biotinylated DNA strand break-mimicking oligonucleotides including DNA_blunt_, DNA_5’P_, DNA_3’P_ and DNA_nick_ were immobilised via streptavidin at the modified surface to study their interaction with PARP1. **b** Real-time monitoring of each step by ATR-FTIR spectroscopy allows to study various parameters: (1) PARP1–DNA-binding kinetics; (2) secondary structure of PARP1 bound to DNA; (3) structural changes of PARP1 upon NAD^+^ addition, kinetics of PARylation and the resulting dissociation of PARP1 from DNA.

Here, we apply a recently developed ATR-FTIR spectroscopic approach combining surface passivation and specific immobilisation^35,36^. By immobilising various biotinylated DNA strand break-mimicking oligonucleotides via streptavidin at the crystal surface, we study the dynamic interplay of PARP1 binding to DNA strand breaks, its subsequent catalytic activation, i.e., PARylation and the subsequent dissociation from DNA (Fig. 1a, b). Time-resolved monitoring enables the direct tracking of the enzymatic reaction and gives access to kinetic parameters as well as structural data on PARP1. We show that PARylation and PARP1 dissociation from DNA are interdependent processes, and that kinetics and efficiencies are determined by the substrate availability and the specific DNA strand break structure. Moreover, we unravel small but distinct structural changes of PARP1 upon addition of NAD^+^, which so far could not be monitored due to the fast catalytic turnover of NAD^+^. Taken together, this study provides direct and holistic insights into the molecular mechanisms underlying PARP1-dependent PARylation in a time-resolved manner.

## Results

### Binding of PARP1 to DNA strand breaks

**Fig. 2:**
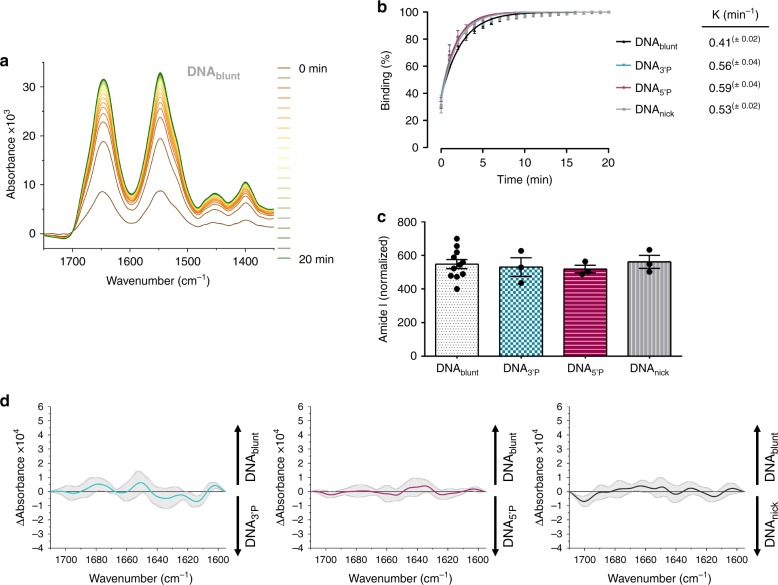
Binding of PARP1 to DNA strand break-mimicking oligonucleotides. **a** Representative time-dependent spectra of PARP1 binding to immobilised DNA_blunt_. **b** Evaluation of time-dependent binding of PARP1 to different types of DNA strand breaks. ‘0 min’ refers to the first time point measured. Signal intensities of amide I bands (1645 cm^−1^) at 20 min were set to 100%. Binding kinetics were calculated via a mono-exponential fit function. **c** Comparison of the amount of PARP1 bound to different types of DNA stand breaks after 20 min of co-incubation. Amide I bands (1645 cm^−1^) were normalised to the amount of immobilised DNA (1220 cm^−1^). **b**, **c** Data from same experiments. Means ± SEM of *n* = 14 (DNA_blunt_) and *n* = 3 (DNA_3’P_, DNA_5’P_, DNA_nick_) independent experiments, respectively. **d** Secondary structure analysis. Difference spectra of amide I bands of PARP1 bound to DNA_blunt_ and DNA_3’P_, DNA_5’P_ or DNA_nick_ were calculated. Average curves and SD (grey) of nine difference spectra are plotted (*n* = 3 independent experiments, respectively). Source data are provided as a Source Data file.

PARP1 can be activated by various types of DNA structures, including single- and double-strand breaks, single-strand overhangs, hairpins and three- or four-way junctions^8,13–16,37^. Recently, it has been shown that while PARP2 and PARP3 are preferentially activated by DNA strand breaks harbouring a 5′ phosphate, PARP1 does not show a clear preference for any type of strand break^8^. To gain more insights into the DNA-binding properties of PARP1, we analysed the interaction of PARP1 with different types of DNA strand break-mimicking oligonucleotides via ATR-FTIR spectroscopy. To this end, we immobilised such biotinylated DNA oligonucleotides containing either a blunt (DNA_blunt_), a 5′ phosphorylated (DNA_5’P_) or a 3′ phosphorylated (DNA_3’P_) double-strand break or a single nick (DNA_nick_) via streptavidin on the modified crystal surface (Fig. 1a). The infrared spectra of the immobilised DNA oligonucleotides displayed the same position of the anti-symmetric phosphate vibration band at 1220 cm^− 1^ (Supplementary Fig. 1b). This band is sensitive to the DNA secondary structure, and indicates that all DNA oligonucleotides exhibit a B-DNA structure^38^. Next, we added PARP1 to the respective immobilised DNA oligonucleotide and monitored the binding process as a function of time. PARP1 signals increased rapidly within the first minute, and reached saturation after ~10 min (Fig. 2a, b; Supplementary Fig. 1d). Calculated rate constants were similar for all DNA oligonucleotides, but binding of PARP1 to DNA_blunt_ was slightly slower. Importantly, PARP1 bound specifically to the immobilised oligonucleotides, as no binding was observed to immobilised streptavidin alone (Supplementary Fig. 1c). We further analysed the amount of PARP1, which was bound to the respective DNA oligonucleotide (Fig. 2c). As K_d_-values of PARP1 binding to DNA oligonucleotides are in the nM range (DNA_blunt_ 7.6 nM and DNA_5’P_ 6.2 nM^39^), a complete saturation of binding sites can be assumed at the applied PARP1 concentration of 2 µM. We did not detect major differences in the amount of bound PARP1, suggesting that the stoichiometry of PARP1–DNA binding is comparable for all tested DNA oligonucleotides. It has been postulated that the binding of PARP1 to DNA strand breaks triggers its allosteric activation leading to structural changes within the HD domain, thereby enhancing accessibility of NAD^+^ to the catalytic cleft^40,41^. To test if different types of DNA strand break structures impact the allosteric activation of PARP1, we analysed the secondary structure of PARP1 bound to the immobilised DNA strand break models. In particular, we generated difference spectra of PARP1 bound to DNA_blunt_, DNA_5’P_, DNA_3’P_ or DNA_nick_, respectively (Fig. 2d). This method eliminates all vibrational modes in the spectra that do not change and thus uncovers explicitly all structural changes taking place. None of the three difference spectra displayed substantial positive or negative bands, suggesting that PARP1 has the same secondary structure when bound to the different DNA structures. In summary, the ATR-FTIR approach allows real-time monitoring of binding of PARP1 to different DNA strand break models. Binding kinetics, stoichiometry and secondary structure of PARP1 appear to be independent of the type of DNA strand break structure.

### Real-time monitoring of PARP1-dependent PARylation

**Fig. 3:**
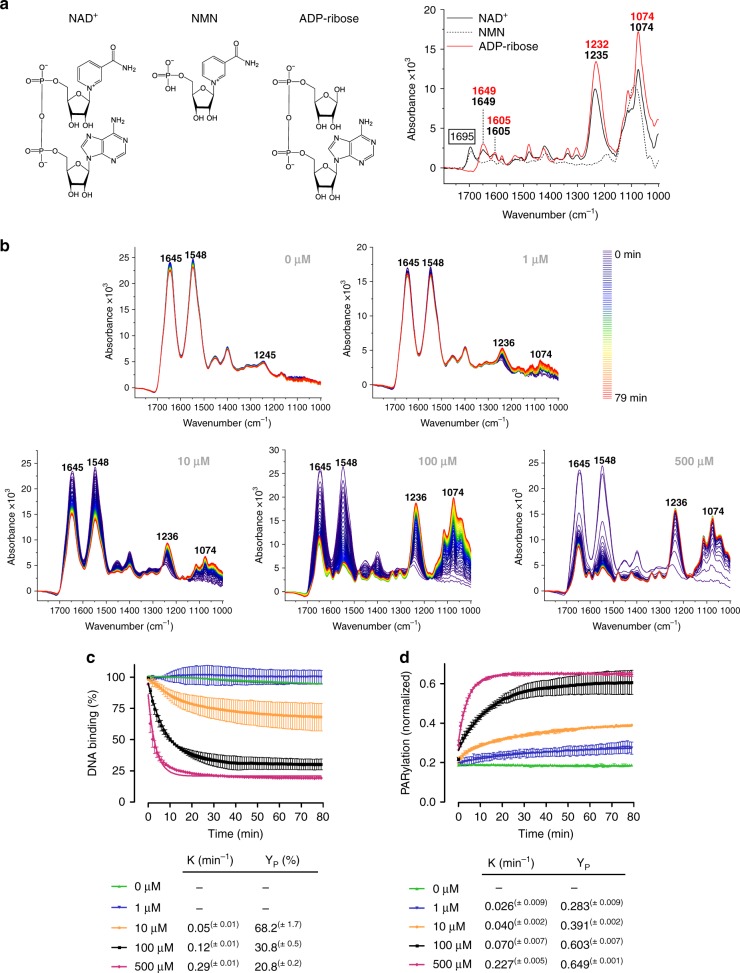
Real-time monitoring of PARP1-dependent PARylation at various NAD^+^ concentrations. **a** Chemical structure and respective IR spectra of NAD^+^, NMN and ADP-ribose. The band assigned to the nicotinamide moiety (1695 cm^−1^) is indicated. **b** Representative time-dependent spectra following the addition of various NAD^+^ concentrations to PARP1 bound to immobilised DNA_blunt_. Amide I (1645 cm^−1^) and amide II (1548 cm^−1^) bands of PARP1 and anti-symmetric (1236 cm^−1^) and symmetric (1074 cm^−1^) phosphate vibrations of generated PAR are indicated. **c**, **d** Evaluation of **b**. ‘0 min’ refers to start of measurements. **c** Dissociation of PARP1 from DNA_blunt_ upon addition of various NAD^+^ concentrations. Time-dependent intensity decrease of the amide II band (1548 cm^−1^) was analysed. Intensity of the amide II band before addition of NAD^+^ was set to 100%. PARP1 dissociation parameters were calculated via a mono-exponential fit function. **d** PAR formation upon addition of various NAD^+^ concentrations. Time-dependent intensity increase of the anti-symmetric phosphate vibration of PAR (1236 cm^−1^) was analysed. Data was normalised to the intensity of the amide II bands (1548 cm^−1^) of PARP1 before addition of NAD^+^. PAR formation parameters were calculated via a mono-exponential fit function. **c**, **d** Data from same experiments. Means ± SEM of *n* = 3 (100 µM) and *n* = 2 (0, 1, 10 and 500 µM) independent experiments, respectively. Source data are provided as a Source Data file.

DNA damage dependent PARylation by PARP1 is a very fast and dynamic process, which includes NAD^+^ binding, PAR synthesis and dissociation of auto-modified PARP1 from DNA^41^. So far, most methods revealed a snapshot of each process, however, detailed insights into the dynamic interplay between the individual processes are still missing. Using the ATR-FTIR spectroscopic approach, we monitored the dynamic process of PARP1-dependent PARylation in real time^35,36^. To lay the foundation for the spectral analysis of the catalytic reaction, we first compared the spectrum of NAD^+^ to the one of ADP-ribose (Fig. 3a), which exhibits an almost identical IR spectrum compared to PAR as shown previously^35^. Both spectra displayed bands at ~1233 and 1074 cm^−1^ corresponding to the anti-symmetric and symmetric phosphate vibrations, as well as bands at 1649 and 1605 cm^−1^, which correspond to the adenine moiety^35,38^. The main difference was observed at the band at 1695 cm^−1^, which is, therefore, indicative of the nicotinamide group. This was confirmed by the spectrum of nicotinamide mononucleotide (NMN), which also displayed a band at 1695 cm^−1^ (Fig. 3a).

To analyse the process of PARylation, PARP1 was allowed to bind to the immobilised DNA oligonucleotides, then NAD^+^ was added, and immediately thereafter IR spectra were recorded for 80 min in 1-min intervals. First, we tested increasing concentrations of NAD^+^ on PARP1 activity when bound to DNA_blunt_ (Fig. 3b). As expected, the spectrum of PARP1 remained nearly constant after adding buffer without NAD^+^. Slight decreases of the amide I and II bands were observed, presumably representing the diffusion of PARP1 from DNA into the supernatant. After adding 1 µM NAD^+^, no major changes in the intensities of the amide I and II bands were observed either, but weak bands at 1236 and 1074 cm^−1^ were detected after 80 min. These can be assigned to the phosphate vibrations of PAR, demonstrating that already at a concentration of 1 µM NAD^+^ a weak modification of PARP1 takes place, which, however, did not result in the dissociation of PARP1 from the DNA_blunt_ oligonucleotide. The application of 10 µM NAD^+^ led to a slight decrease of the amide I and II bands and at the same time a stronger increase of the PAR-derived phosphate vibration bands. This indicates that PARP1 was partly released from DNA, while at the same time PAR was formed. Both effects became stronger with increasing NAD^+^ concentrations. At a NAD^+^ concentration of 500 µM, already after ~10 min the PARP1 signal disappeared almost completely. Comparison of the dissociation of PARP1 from DNA_blunt_ at different NAD^+^ concentrations indicate that higher NAD^+^ concentrations result in a more efficient dissociation of PARP1 from DNA (Fig. 3b, c). The calculated rate constants revealed that at higher NAD^+^ concentrations not only the PARP1 dissociation became more efficient but this process was also significantly accelerated (Fig. 3c). Therefore, the catalytic turnover of NAD^+^ appears to be directly connected to the dissociation of PARP1 from the DNA_blunt_ molecules. Interestingly, the PAR signal at the crystal surface increased over time, even though PARP1 was simultaneously released. This formation of PAR was more pronounced and faster at higher NAD^+^ concentrations (Fig. 3d). To test if this PAR signal was caused by residual DNA-bound auto-modified PARP1, the crystal surface was washed with 1 M NaCl directly after the PARylation reaction to disrupt ionic interactions, and thereafter, with 1% SDS to disrupt non-covalent interactions (*N.B*. streptavidin–biotin interactions are not disrupted at this SDS concentration^42,43^). Washing with 1 M NaCl did not change the spectra significantly, suggesting that ionic interactions do not contribute primarily to the DNA_blunt_–PARP1 interactions (Supplementary Fig. 2a, c). Notably, under conditions without NAD^+^, more PARP1 could be removed by the high ionic strength buffer in comparison with conditions with 1 µM NAD^+^. This suggests that already minor PARylation impacts the PARP1–DNA interaction. After washing with 1% SDS, most PARP1 was removed from the crystal, while the PAR signal was still detectable (Supplementary Fig. 2a, b). The ratio of the anti-symmetric phosphate vibration (1236 cm^−1^) and the amide II band of PARP1 (1548 cm^−1^) can be used to estimate the PAR to protein ratio at the crystal surface. At higher NAD^+^ concentrations, this ratio increased after washing with SDS buffer, suggesting that more PARP1 than PAR could be removed from the crystal surface (Supplementary Fig. 2d). These data imply that besides PARP1 itself, either DNA and/or streptavidin, which are immobilised at the crystal surface, became PARylated. As recently reported, covalent PARylation of DNA termini may represent one possible explanation for the PAR signal observed at the crystal surface^44–47^. In addition, streptavidin represents a potential PARylation target. This may occur, in a mechanism similar to the one recently demonstrated, showing that the substrate specificity of PARP1 is mainly determined by substrate proximity, which can lead to the PARylation of noncanonical substrates, such as GST^48^. Before addressing this issue again (see below), we analysed unbound PARP1 in the supernatant after completion of the PARylation reaction via western blotting to test the PARylation status of PARP1 released from the DNA oligonucleotides. Interestingly, the majority of PARP1 was strongly auto-modified (Supplementary Fig. 2e). In summary, the presence of NAD^+^ as a PARP1 substrate led to a dose-dependent ‘trans-PARylation’ of molecules at the crystal surface, which correlated positively with the dissociation kinetics of PARP1 from DNA.

**Fig. 4:**
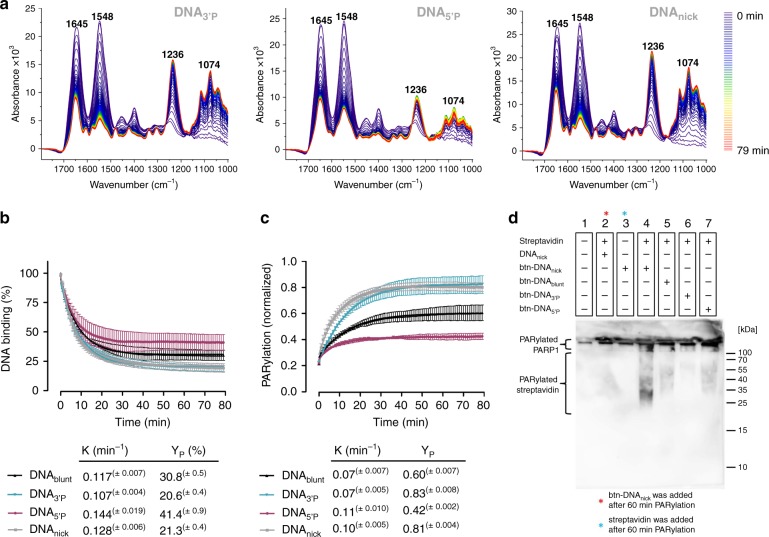
Real-time monitoring of PARP1-dependent PARylation at various DNA strand break models. **a** Representative time-dependent spectra following the addition of 100 µM NAD^+^ to PARP1 bound to immobilised DNA_3’P_, DNA_5’P_ or DNA_nick_. Amide I (1645 cm^−1^) and amide II (1548 cm^−1^) bands of PARP1 and anti-symmetric (1236 cm^−1^) and symmetric (1074 cm^−1^) phosphate vibrations of generated PAR are indicated. **b**, **c** Evaluation of **a**. ‘0 min’ refers to start of measurements. **b** Dissociation of PARP1 from DNA_blunt_, DNA_3’P_, DNA_5’P_ or DNA_nick_ upon addition of NAD^+^. Time-dependent intensity decrease of the amide II band (1548 cm^−1^) was analysed. Intensity of the amide II band before addition of NAD^+^ was set to 100%. PARP1 dissociation parameters were calculated via a mono-exponential fit function. **c** PAR formation upon addition of NAD^+^. Time-dependent intensity increase of the anti-symmetric phosphate vibration of PAR (1236 cm^−1^) was analysed. Data were normalised to the intensity of the amide II bands (1548 cm^−1^) of PARP1 before addition of NAD^+^. PAR formation parameters were calculated via a mono-exponential fit function. **b**, **c** Data from same experiments. Means ±  SEM of *n* = 3 independent experiments. **d** Analysis of covalent PARylation of streptavidin in the presence of biotinylated (btn-DNA) and non-biotinylated DNA (DNA) via western blot and subsequent immunodetection of PAR. PARP1 (1 µM) and NAD^+^ (500 µM) were present in all samples. Immunodetection of PARP1 is shown in Supplementary Fig. 3e. Source data are provided as a Source Data file.

Next, we tested the impact of the type of DNA strand break structure on the PARylation process. After PARP1 bound to DNA_3’P_, DNA_5’P_ and DNA_nick_, PARylation was started by adding 100 µM NAD^+^. Depending on the individual DNA strand break model, significant differences were observed (Fig. 4a–c). Thus, PARP1 dissociated more efficiently from DNA_3’P_ and DNA_nick_, and less efficiently from DNA_5’P_ compared to DNA_blunt_ (Fig. 4b). This correlated with a more pronounced PAR formation at DNA_3’P_ and DNA_nick_, and a lower PAR formation at DNA_5’P_ compared to DNA_blunt_ (Fig. 4c). The kinetics of PARP1 dissociation from DNA differed only moderately among the different DNA structures, but correlated with the kinetics of PAR formation (Fig. 4b, c). After washing with 1% SDS, most PARP1 was removed from the crystal, whereas the PAR signal remained largely constant (Supplementary Fig. 3a–c). These data again suggest that the PAR signal detected did not derive from residual auto-modified PARP1 at the crystal surface, but from trans-PARylation of DNA or streptavidin. Therefore, we tested the potential trans-PARylation of streptavidin and/or DNA in gel-based assays. Notably, not DNA, but streptavidin was identified as a target for covalent modification (Fig. 4d; Supplementary Fig. 3e, f). In accordance with previous data^48^, this covalent modification was mediated by close proximity between PARP1 and streptavidin, which only occurred in the presence of biotinylated DNA and not in the presence of non-biotinylated DNA.

Next, we compared the ATR-FTIR spectroscopic findings on PARP1 activation by different DNA oligonucleotides with an orthogonal approach using a conventional gel-based assay in combination with TAMRA-labelled NAD^+^ (Supplementary Fig. 3d). In accordance with the IR spectroscopic findings, PARylation of PARP1 activated by DNA_5’P_ was significantly reduced. In contrast, we did not detect significant differences between PARylation of PARP1 activated by DNA_blunt_, DNA_3’P_ and DNA_nick_, probably due to a lack of sensitivity of the gel-based assay. Those data demonstrate the high sensitivity and reproducibility of the ATR-FTIR spectroscopic approach in comparison with conventional biochemical assays. In summary, these data provide strong evidence that streptavidin serves as a noncanonical PARylation substrate at the crystal surface, which can be efficiently used as a read-out for the analyses of PARP1 activity. Furthermore, these data demonstrate that PARylation by PARP1 is dependent on the type of DNA strand break structure affecting the dynamic and strongly interdependent processes of PARylation and PARP1 dissociation from DNA.

### Structural changes of PARP1 upon PARylation

**Fig. 5:**
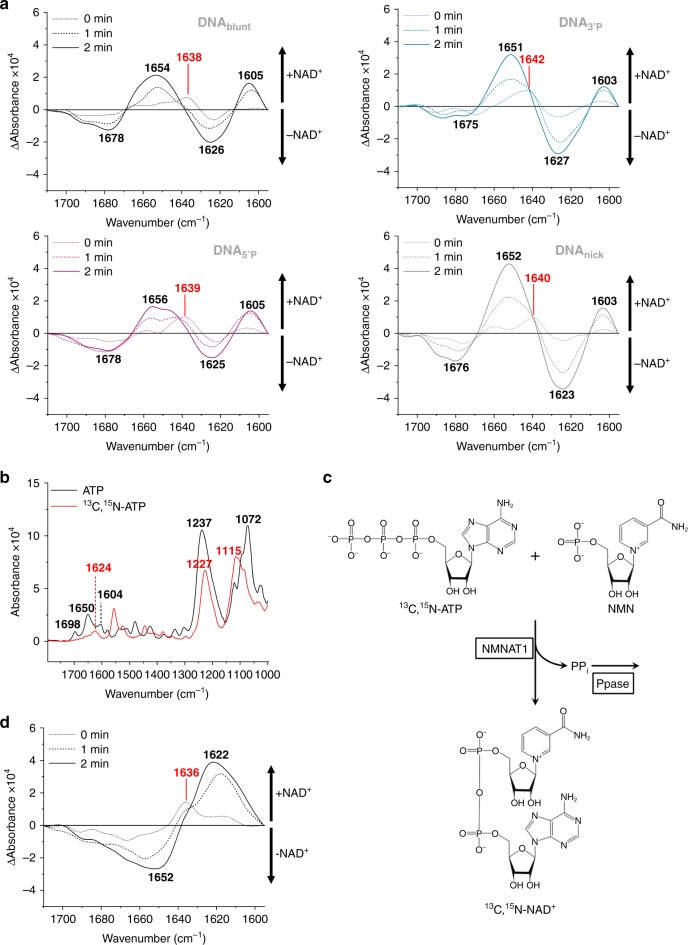
Secondary structure analysis of PARP1 upon NAD^+^ addition. **a** Comparison of PARP1 bound to immobilised DNA_blunt_, DNA_3’P_, DNA_5’P_ or DNA_nick_. Difference spectra of amide I bands of PARP1 before and after the addition of 100 µM NAD^+^ (0, 1 and 2 min) were calculated. Average curves of *n* = 3 independent experiments are plotted, respectively. SDs are shown in Supplementary Fig. 4a. Positive difference bands distinct from PAR bands are indicated in red. **b** Spectra of unlabelled and isotopically labelled ^13^C,^15^N-ATP. **c** Strategy for the enzymatic synthesis of isotopically labelled ^13^C,^15^N-NAD^+^. **d** Difference spectra of amide I bands of PARP1 bound to DNA_blunt_ before and after the addition of 100 µM ^13^C,^15^N-NAD^+^ (0, 1 and 2 min) were calculated. Average curves of *n* = 3 independent experiments are plotted. SDs are shown in Supplementary Fig. 4d. The positive difference band distinct from the PAR band is indicated in red. Source data are provided as a Source Data file.

Several approaches demonstrated that PARP1 undergoes allosteric activation upon DNA binding, which is connected to structural distortions in the HD domain^14,22,27,28,41^. By using a non-hydrolysable NAD^+^ analogue, recently first evidence was provided that NAD^+^ binding to the catalytic centre might result in reverse allostery of PARP1 with regards to DNA binding^27^. The authors suggested that NAD^+^ binding further shifts the HD to the unfolded conformation, which can then induce changes in the binding affinity for DNA lesions. However, due to the fast catalytic turnover of NAD^+^ by PARP1, direct evidence supporting this hypothesis is still missing. Furthermore, it is still unknown, how PARylation affects the secondary structure of PARP1. To address these questions, we analysed structural changes of PARP1 during PARylation by reaction-induced difference spectroscopy. Therefore, we calculated difference spectra of PARP1 bound to the different DNA oligonucleotides, before and after the addition of 100 µM NAD^+^ (Fig. 5a; Supplementary Fig. 4a). All negative bands of the difference spectra represent structures that were more pronounced before the addition of NAD^+^, and all positive bands represent structures that were more pronounced after the addition of NAD^+^. The spectrum of PAR reveals two bands (1649 and 1605 cm^−1^) within the amide I region of PARP1 (1710–1595 cm^−1^) (Fig. 3a). Since during PARylation the PARP1 signal decreased and the overlapping PAR signal increased, we limited the structural analysis to the first 2 min. Spectral characteristics of PARP1 were found to be comparable for all DNA oligonucleotides, indicating that structural changes of PARP1 are independent of the type of DNA strand break. Two minutes after NAD^+^ addition, distinct positive bands at ~1655 cm^−1^ and ~1605 cm^−1^ were detected, which can be assigned to the overlapping PAR bands. This implies that already at very early time points of the PARylation reaction difference spectra are dominated by the arising PAR chains. To compensate the decrease of the PARP1 signal due to dissociation during PARylation, all amide I bands were normalised before calculating difference spectra. The formation of PAR, however, resulted in additional absorbance in the amide I region, which could not be considered in the normalisation procedure. Consequently, all positive difference bands arising from PAR within the difference spectra also resulted in negative difference bands. Those bands were therefore excluded from secondary structure predictions. Despite the strong impact of PAR on the difference spectra, we detected a positive band at 1639–1642 cm^−1^ directly after the addition of NAD^+^ (Fig. 5a; Supplementary Fig. 4a). This band position is neither typical for PAR nor for NAD^+^, and is therefore indicative for changes in the PARP1 secondary structure. Isotopic labelling of molecules offers a possibility to induce distinct frequency shifts of infrared absorption bands. By introducing isotopic labels into NAD^+^, we aimed to shift the bands at 1649 and 1605 cm^−1^, which overlap the amide I region, to lower frequencies, thereby improving structural analysis of PARP1 during PARylation. We synthesised isotopically labelled NAD^+^ enzymatically using ^13^C,^15^N-ATP. First, we analysed the infrared spectrum of ^13^C,^15^N-ATP and compared it to the spectrum of unlabelled ATP (Fig. 5b). As expected, the infrared absorption bands of ATP at 1650 and 1604 cm^−1^ were shifted to lower frequencies by the introduced isotopic labels. One band at 1624 cm^−1^ remained within the amide I region. Yet, an improved analysis of structural changes of PARP1 was expected. The enzymatic synthesis of isotopically labelled NAD^+^ was performed using nicotinamide nucleotide adenylyltransferase 1 (NMNAT1), which uses ATP and NMN as substrates, while releasing pyrophosphate as a by-product. To shift the equilibrium of the reaction towards the production of NAD^+^, we added pyrophosphatase (PPase), which cleaves the pyrophosphate produced (Fig. 5c). This approach resulted in an almost complete conversion of ^13^C,^15^N-ATP and NMN to ^13^C,^15^N-NAD^+^ (Supplementary Fig. 4b). Next, we analysed PARylation by the addition of 100 µM ^13^C,^15^N-NAD^+^ to PARP1 bound to immobilised DNA_blunt_. As expected, the PARylation reaction was not affected by the isotopic label (Supplementary Fig. 4c). Amide I and II bands decreased rapidly, while distinct bands at 1233 and 1078 cm^−1^ arose, which represent the slightly shifted phosphate vibrations. Calculated difference spectra revealed a clear positive band at 1622 cm^−1^ after 2 min (Fig. 5d; Supplementary Fig. 4d). This band can be assigned to isotopically labelled PAR, which displays similar infrared absorption bands like isotopically labelled ATP (Fig. 5b). Strikingly, directly after the addition of ^13^C,^15^N-NAD^+^, a distinct positive band at 1636 cm^−1^ was resolved similar to the one observed after the addition of unlabelled NAD^+^ (Fig. 5d; Supplementary Fig. 4d). Since this effect was independent of the NAD^+^ substrate, these data provide strong evidence for structural changes of PARP1 itself. The position of the band at 1636–1642 cm^−1^ can be assigned to disordered or β-sheet structures suggesting that the proportion of those structures increases after addition of NAD^+^ ^49^. Mainly two processes could cause these structural changes: NAD^+^ binding or PARylation of PARP1 itself, which, at this level, did not result in its dissociation from DNA. To test the second hypothesis, we performed spectral analysis of PARP1 during PARylation at various NAD^+^ concentrations (Supplementary Fig. 5). Low concentrations of NAD^+^, which resulted in only slight PARylation, did not reveal significant structural changes of PARP1, even after 60 min. In contrast, directly after the addition of 500 µM NAD^+^ a clear positive difference band at 1640 cm^−1^ could be resolved. These observations suggest that the structural changes are caused by binding of NAD^+^ to the catalytic centre, which were only resolved at high NAD^+^ concentrations, when binding occurred simultaneously at most PARP1 molecules.

To investigate the effect of NAD^+^ binding in more detail, we studied the PARP1 variant PARP1^E988K^. The residue E988 is highly conserved among PARPs, and is part of the catalytic triad within the donor site^50^. Mutations of this residue were shown to result in MARylation activity only^51–54^. Accordingly, only elongation, but not NAD^+^ binding, seems to be affected by these mutations. First, we analysed the activity of PARP1^E988K^ in a gel-based assay using TAMRA-labelled NAD^+^ (Supplementary Fig. 6a). This analysis confirmed the MARylation activity of PARP1^E988K^. Thus, instead of an upwards shift of PARP1^E988K^ in the gel, which is caused by PARP1 automodification with long PAR chains, a clear increase of the intensity of the PARP1^E988K^ band was evident, indicating automodification with monomers. Next, we tested the DNA-binding properties of PARP1^E988K^ via ATR-FTIR spectroscopy. Therefore, we immobilised DNA_blunt_ at the crystal surface, added PARP1^E988K^ and monitored binding over time (Supplementary Fig. 6b). PARP1^E988K^ signal intensities increased rapidly and reached saturation ~10 min after addition, which is comparable with wild-type PARP1. Interestingly, binding of PARP1^E988K^ (0.52 min^−1^) was slightly faster compared to wild-type PARP1 (0.41 min^−1^). Yet, the total amount of PARP1^E988K^, which was bound to DNA_blunt_, was 25% less (Supplementary Fig. 6c). So far, no structural information about PARP1 E988 mutants exist. We calculated difference spectra to compare secondary structures of wild-type PARP1 and PARP1^E988K^ when bound to DNA_blunt_. Interestingly, distinct positive and negative bands were resolved, indicating distinct structural differences between both variants (Supplementary Fig. 6d). Two positive difference bands at 1660 and 1637 cm^−1^ were resolved representing structural components, which were more frequent in PARP1^E988K^. The band at 1660 cm^−1^ is indicative for α-helices, β-turns or 3_10_-helices, while the band at 1637 cm^−1^ is indicative of β-sheet structures^49,55^. In addition, one major negative band at 1612 cm^−1^ was resolved, representing structural components, which were more frequent in wild-type PARP1. The positions of this band can also be assigned to β-sheet structures^49,55^. A position at lower frequencies can be the result of stronger hydrogen bonds and an increase in the number of β-strands^49,56,57^. In turn, this suggests that the introduced mutation results in the loss of compact or extended β-sheet structures. Considering that the residue E988 is part of a β-sheet structure within the catalytic domain, these results suggest a destabilising effect of the catalytic domain caused by the introduced mutation. Next, we tested the effect of NAD^+^ addition on the structure of the PARP1^E988K^ mutant (Supplementary Fig. 6e). Neither PAR formation nor PARP1 release was observed, confirming the loss of PARylation activity and trapping of PARP1 molecules at the DNA oligonucleotides. Similarly to wild-type PARP1, distinct structural changes were also observed for PARP1^E988K^ upon addition of NAD^+^ (Supplementary Fig. 6f). Of note, difference spectra, which are much more sensitive than absorbance spectra, did not reveal PAR signals after NAD^+^ addition, indicating a complete absence of PAR formation. Directly after NAD^+^ addition, one major positive difference band at 1649 cm^−1^ was resolved. It represents structures, which were more frequent after NAD^+^ addition and can be assigned to α-helices or disordered structures^49,55^. Those structural changes were only resolved directly after NAD^+^ addition, and are therefore very likely caused by NAD^+^ binding rather than by MARylation. Notably, this positive difference band was close to the position of the positive difference band observed for PARP1 after NAD^+^ addition (1639–1642 cm^−1^). As the complete spectral region can be assigned to disordered structures, those data provide further evidence for a shift of the HD to the unfolded conformation upon NAD^+^ binding, which has been postulated before^27^. As the structure of PARP1^E988K^ was significantly affected by the introduced mutation (Supplementary Fig. 6d), it is very likely that the NAD^+^-binding mechanism and structural consequences thereof were also affected, which may explain why the exact positions of the difference bands were not identical for PARP1^WT^ and PARP1^E988K^. In summary, we provide evidence for distinct changes in the secondary structure of PARP1 upon binding to NAD^+^, suggesting the formation of disordered or β-sheet structures.

### Structural changes of PARP1 upon inhibitor binding

**Fig. 6:**
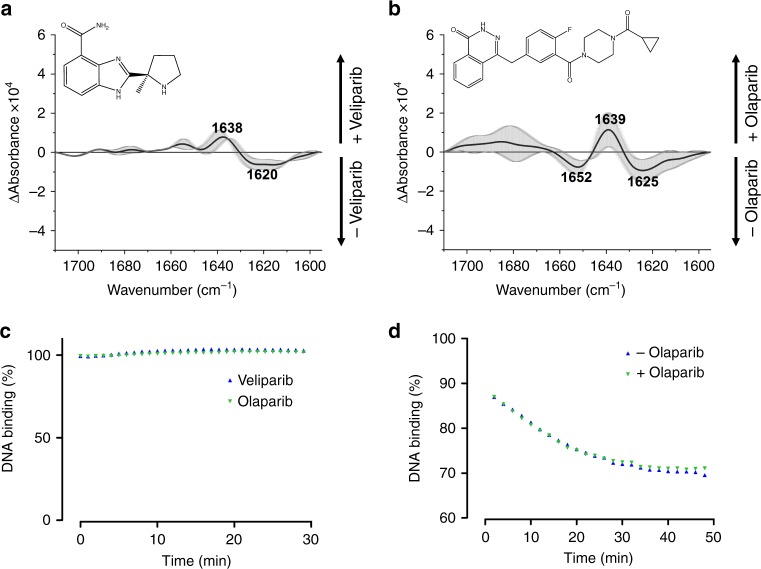
Effect of inhibitor binding on PARP1 structure and DNA binding properties. **a** Secondary structure analysis of PARP1 upon binding to veliparib. Difference spectra of amide I bands of PARP1 bound to DNA_blunt_ were calculated before and after the addition of 10 µM veliparib. Average curves and SDs (grey) of two experiments are plotted. **b** Secondary structure analysis of PARP1 upon binding to olaparib. Difference spectra of amide I bands of PARP1 bound to DNA_blunt_ before and after the addition of 10 µM veliparib were calculated. Average curves and SDs (grey) of two experiments are plotted. **c** Evaluation of inhibitory effect of veliparib and olaparib. Dissociation of PARP1 from DNA_blunt_ upon addition of 100 µM NAD^+^ in the presence of inhibitor was tested. Time-dependent intensity decrease of the amide II band (1548 cm^−1^) was analysed. Intensity of the amide II band before addition of NAD^+^ was set to 100%. **d** Analysis of ‘trapping’ effect of olaparib. Binding of PARP1 to immobilised DNA_blunt_ was competed by the addition of free DNA_blunt_ in the supernatant in the absence or in the presence of olaparib. Time-dependent intensity decrease of the amide I band (1645 cm^−1^) was analysed. Intensity of the amide I band before addition of free DNA_blunt_ was set to 100%. Source data are provided as a Source Data file.

The mechanism of reverse allostery of PARP1 with regards to its DNA-binding affinity has not only been proposed for NAD^+^ binding but also for the binding of PARP inhibitors^9,58,59^. Such allosteric conformational changes were expected to stabilise DNA binding of PARP1 and could thereby explain the so called ‘trapping’ mechanism^58,59^, which is considered to be responsible for the clinical efficacy of PARP inhibitors. To analyse the proposed allosteric binding, we added veliparib and olaparib to DNA_blunt_-bound PARP1 and performed a secondary structure analysis. We calculated difference spectra of PARP1 in the presence or absence of inhibitors (Fig. 6a, b). Positive difference bands represent structural components of the inhibitor-bound state, and negative difference bands represent structural components of the unbound state. Interestingly, binding of veliparib and olaparib resulted in a positive band at ~1639 cm^−1^. The position of the band was similar to the one observed after addition of NAD^+^ (Fig. 5a, d) and can be assigned to disordered or β-sheet structures^49^. This finding points to a common binding mechanism with similar structural consequences for PARP1. Moreover, a negative band was observed in the region 1620–1625 cm^−1^, which can be assigned to β-sheet structures as well^49^. In contrast to the position at ~1639 cm^−1^, the position at 1620–1625 cm^−1^ is more representative for extended β-sheet structures with stronger hydrogen bonds^49,56,57^. This suggests a loss of this type of β-sheet structures upon binding to PARP inhibitors. Next, we tested the inhibitory effect of both inhibitors by adding 100 µM NAD^+^. The presence of veliparib or olaparib completely suppressed the formation of PAR and thereby the dissociation of PARP1 from DNA (Fig. 6c; Supplementary Fig. 7a, b). Importantly, difference spectra did not reveal significant structural changes of PARP1 upon NAD^+^ addition after preincubation with inhibitors (Supplementary Fig. 7a, b). This demonstrates the specificity of the observed structural changes of PARP1 after binding of molecules to the catalytic cleft. As the mechanism of allosteric binding was proposed to stabilise DNA binding, we tested the effect of inhibitor on PARP1/DNA_blunt_ interaction in a competition assay. We focused our analysis to olaparib, as the trapping effect was shown to be stronger for this inhibitor^9^. PARP1 was bound to immobilised DNA_blunt_, and free DNA_blunt_ was added in the supernatant in the absence or presence of olaparib. The signal of PARP1 decreased independent of the presence of olaparib (Fig. 6d). This indicates that olaparib did not stabilise PARP1 on immobilised DNA_blunt_, but allowed an exchange of PARP1 to the free DNA_blunt_ in the supernatant. Similar results have been obtained in previous studies by applying a series of biochemical methods^1,60^. In summary, binding of veliparib and olaparib to PARP1 result in structural changes of PARP1, which are similar to those of NAD^+^ binding. Binding did not lead to stronger DNA-binding affinity, yet was able to trap PARP1 on DNA.

## Discussion

**Fig. 7:**
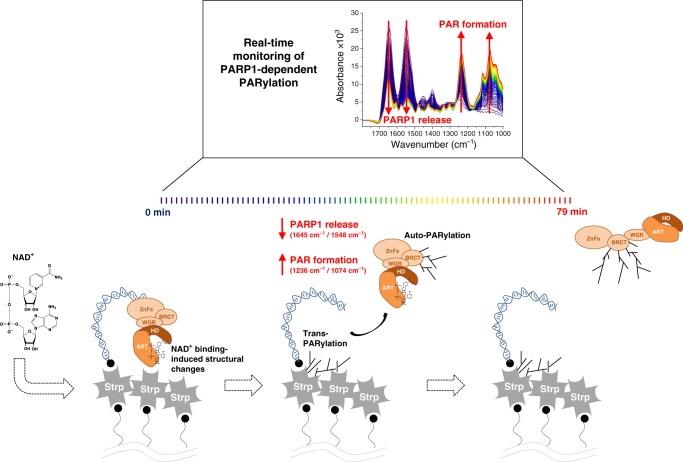
Mechanistic model of PARP1-dependent PARylation derived by time-resolved ATR-FTIR spectroscopy. Directly after NAD^+^ addition binding of NAD^+^ to the catalytic centre induces distinct structural changes of PARP1. This is followed by the release of PARP1 from DNA, which is reflected by a decrease of PARP1 signal intensity (amide I (1645 cm^−1^) and amide II (1548 cm^−1^) bands), and the simultaneous formation of PAR, which is reflected by an increase of PAR signal intensities [anti-symmetric (1236 cm^−1^) and symmetric (1074 cm^−1^) phosphate vibrations]. The observed formation of PAR is mainly due to trans-PARylation of streptavidin, which is mediated by the close proximity of streptavidin to the DNA strand break.

In this study, we investigated the molecular dynamics of DNA-dependent, PARP1-mediated PARylation in real time by direct recording of ATR-FTIR spectroscopic data (Fig. 7). While binding and activation by PARP1 showed similar kinetics at four different DNA strand break structures, distinct differences were revealed for the PARylation reaction itself, as well as for the dissociation of PARP1 from the different DNA molecules. A strong PAR formation correlated with an efficient dissociation of PARP1 from DNA demonstrating the interdependence of both processes. Interestingly, the strongest PAR formation and most efficient dissociation were observed at single nicks (DNA_nick_) and 3′ phosphorylated ends (DNA_3’P_). The strong activation at single nicks is consistent with the fact that PARP1 is of critical importance for the rapid and efficient repair of single-strand breaks^2,61,62^. Considering PARP1’s general role in DNA repair, the preferred activation of PARP1 at 3′ phosphorylated ends is not unexpected either. 3′ phosphorylated ends are non-physiological and occur frequently during genotoxic insults in cells. Besides the direct induction via ionising irradiation they can occur after topoisomerase 1 inhibition or during base excision repair^63^. Accordingly, we observed a weaker activation of PARP1 at 5′ phosphorylated DNA ends, which mainly occur under physiological conditions during DNA replication. Therefore, our data support PARP1’s central role in the DNA damage response. Interestingly, PARP2 and PARP3 are mainly active at 5′ phosphorylated ends^8^, suggesting specific roles for the DNA-dependent PARPs in cells. Nevertheless, the fact that PARP1 was active at all tested DNA strand break structures and that differences in PARylation activities at the different DNA oligonucleotides were less than twofold is consistent with an assumed redundancy between PARP1 and PARP2^64^. While each of them might have preferential DNA structures for activation, and thereby fulfil unique roles in cellular processes, losing one of them can at least partly be compensated by the other. It is worth mentioning that recent data analysing the activation of PARP1 at various types of DNA strand breaks via a colorimetric assay did not result in significant differences in PAR formation^8^. Still, there was a trend of preferred activation of PARP1 at 3′ phosphorylated ends, which demonstrates the high sensitivity and reproducibility of the IR spectroscopic approach in comparison with conventional methods. While most methods detect PAR formation only indirectly (e.g. via antibodies or streptavidin–biotin interaction), the ATR-FTIR spectroscopic approach enables direct and label-free monitoring of the enzymatic reaction, thus improving sensitivity, reproducibility and time-resolved analyses.

After adding NAD^+^, we observed a decrease of PARP1 signal indicating the dissociation of PARP1 from DNA due to PARylation. It is generally assumed that automodification of PARP1 results in steric and electrostatic repulsion and thereby the release of PARP1 from DNA^8,11^. Yet, the exact mechanism is still not completely understood. Our approach allows to study the interplay of PARP1-mediated PARylation and its dissociation from DNA in real time. Remarkably, along with PARP1’s dissociation, we observed an increase of PAR signal at the crystal surface, which persisted after removal of PARP1 and which is caused by the covalent modification of streptavidin. Even though streptavidin is not a bona fide target of PARP1, the proximity between PARP1 and streptavidin in our setup apparently allows covalent modification, in an analogous way as shown for GST^48^. Considering DNA damage-dependent PARylation in cells, a similar mechanism of trans-PARylation might explain the observed modification of histones^2,5,65^.

The strong positive correlation of PAR formation and PARP1 dissociation suggests that the trans-PARylation supports PARP1 dissociation. Yet, since kinetics of dissociation were faster than kinetics of PAR formation, it is likely that automodification also contributes to the dissociation process. If automodification of PARP1 mediates its dissociation from DNA, we would expect a fast increase of the PAR signal, which decreases along with the dissociation of auto-modified PARP1. However, a decrease of PAR signal was not observed at any time. This might be caused by limited detectability, because the trans-PARylation at the crystal surface overlays the decrease of the PAR signal, due to the release of auto-modified PARP1. Still, the absence of a detectable decrease of the PAR signal suggests that there is no strong automodification of PARP1 occurring at the crystal surface. Therefore, it can be assumed that already weak automodification of PARP1 mediates an efficient release from DNA. This is in line with findings that PARP3 can dissociate from DNA even though it produces only very short chains^8^. Notably, as we detected strong automodification of PARP1 after its release from the crystal surface, but not or only to a minor extent at the crystal surface, this leaves room for speculation that PARP1 automodification mainly occurs after its initial activation in the absence of DNA.

Activation of PARP1 upon DNA binding can be explained by an allosteric binding mechanism resulting in the partial unfolding of the HD domain and thereby accessibility of NAD^+^ to the active site. In contrast, structural consequences of NAD^+^ binding and PARP1 automodification are less well understood, mainly due to the dynamic and complex interdependencies of these processes. By studying PARP1-dependent PARylation in real time, we uncovered structural changes of full-length PARP1 upon addition of NAD^+^. The observed positive difference band between 1636–1642 cm^−1^ can be assigned to an increase of disordered or β-sheet structures. As those structural changes were only observed directly after addition of NAD^+^, in the absence of significant PAR signal and only at high NAD^+^ concentrations, we propose that they are rather related to NAD^+^ binding and not to automodification with PAR. This hypothesis is further supported by the results obtained with the PARP1^E988K^ mutant. Interestingly, a recent study revealed a further shift of the HD towards the unfolded state upon binding of a non-hydrolysable NAD^+^ analogue^27^. Those structural changes were linked to an increased affinity of PARP1 for DNA damage. Our data support those findings and suggest an increase of disordered structures probably linked to the unfolding of the HD domain upon NAD^+^ binding. Yet, we did not observe increased affinity to DNA upon NAD^+^ binding, but instead, the immediate dissociation of PARP1 from DNA. Even though a stabilising effect directly after NAD^+^ binding might still be possible, which was then immediately reversed by the following catalytic reaction, the observed stabilising effect might also be specifically caused by the NAD^+^ analogue used by Langelier et al.^27^, as this might have a slightly different binding mechanisms than naturally occurring NAD^+^. The binding mechanisms of NAD^+^ causing additional destabilization of the HD domain might be a key step for further activation of PARP1 and PAR elongation. This mechanism could explain why PAR elongation rates remarkably exceed initiation rates^50^.

Interestingly, we observed similar structural changes of PARP1 upon addition of the PARP inhibitors. This suggests that, besides NAD^+^ binding, also PARP inhibitor binding results in a destabilization of the HD. Whereas inhibitors mimic the nicotinamide moiety of NAD^+^ and solely bind to the nicotinamide-binding pocket within the ART domain of PARP1, NAD^+^ also binds to the phosphate and adenine–ribose-binding site. The impact on PARP1’s structure was more pronounced for olaparib compared with veliparib, which is the more potent inhibitor and the sterically more demanding molecule. Therefore, our data suggest that binding of molecules to the nicotinamide-binding pocket is sufficient for structural changes of PARP1, but is likely to depend on their size and binding strength.

In our ATR-FTIR spectroscopic approach, DNA strand break models were immobilised via streptavidin at the crystal surface. In principle, streptavidin may undergo structural changes during measurements as well, which could not be distinguished from structural changes of PARP1. However, such changes are rather unlikely, since it is known that streptavidin is an extremely stable protein^66^, and we reported previously that even elevated temperatures or high concentrations of detergents did not result in significant structural changes of streptavidin^36^. Furthermore, we can exclude that PARylation of streptavidin induced the structural changes at the band position ~1640 cm^−1^, since changes at this band position were also observed upon application of PARP inhibitors instead of NAD^+^. Finally, the controls included in this study, e.g. analysis of potential NAD^+^-induced effects in the presence of PARP inhibitor or analysis of PARP1^E988K^, provide strong evidence for PARP1-specific effects.

The concept of a PARP1 trapping mechanism is based on findings showing that PARP1 catalytic inhibition showed higher cytotoxicity than PARP1 depletion. The trapped PARP1 complex at DNA lesions prevents DNA metabolic processes and thereby induces cell death^59^. The mechanism behind this is still controversial. Two non-mutually exclusive hypotheses exist: on the one hand, it is suggested that an allosteric binding mechanism increases PARP1’s affinity for DNA^9,67^, on the other hand, the mere inhibition of catalytic activity, which inhibits PARylation- dependent dissociation, may stabilise PARP1 on DNA^1,60^. Performing a competitive DNA-binding experiment, we provide evidence for the second hypothesis, i.e. while DNA binding itself was not affected by the presence of inhibitor, inhibition of PARP1 activity prevented the resulting dissociation of PARP1 from DNA.

In conclusion, this study provides insights into the dynamic and overlapping processes of PARP1-dependent PARylation, including activation at DNA strand breaks, NAD^+^ binding, PARylation and dissociation of PARP1 from DNA. The direct and label-free spectroscopic tracking of the enzymatic reaction enabled high sensitivity and reproducibility, and revealed information on kinetics as well as on the tightly controlled structural changes of PARP1. Since PARP1 is a promising target for precision therapy by selectively treating DNA repair-deficient cancers, the detailed examination of the underlying molecular processes of PARP1-dependent PARylation can give us a starting point to improve our methodologies and develop future therapies. Finally, the ATR-FTIR spectroscopic approach not only provides a platform to study molecular processes of PARP1-dependent PARylation but has also the potential to unravel the enzymatic mechanisms of other enzymes involved in DNA metabolism as well as to provide a deeper understanding of how post-translational modifications in general control enzymatic functions and protein structures.

## Methods

### Purification of PARP1

PARP1 was purified as described previously^35,68^, with some modifications. Briefly, bacterial pellets [*E. coli* strain Rosetta 2 (DE3)] from 2 l cultures were resuspended in lysis buffer (25 mM HEPES pH 8.0, 500 mM NaCl, 0.5 mM DTT, 10 mM benzamide), supplemented with 0.1% NP-40, Complete EDTA-free protease inhibitor cocktail (Roche) and 1 mg/ml lysozyme (Sigma-Aldrich), and were sonicated four times for 20 s each. Then, 5 µg/ml DNase I (Roche) was added and the lysate was incubated for 1 h. Cell debris was removed by centrifugation (68,000×*g* for 2 h), the supernatant was filtered through a 0.2-µm syringe filter (Corning) and loaded onto a HisTrap HPcolumn (GE Healthcare). After washing with 10 ml of 1 M NaCl, PARP1 was eluted with 30 ml of 500 mM imidazole. The elution fraction was diluted to a final NaCl concentration of 375 mM with no-salt heparin buffer (50 mM Na-phosphate pH 7.0; 1 mM EDTA) and loaded onto a heparin HP column (GE Healthcare). PARP1 was eluted by gradually increasing the NaCl concentration up to 1 M (30 ml). PARP1 containing fractions were concentrated and buffer was exchanged (50 mM Tris pH 8, 150 mM NaCl, 0.5 mM DTT) via centrifugal filters (Amicon Ultra 15, 10 kDa MWCO). PARP1 was further purified by size-exclusion chromatography using a HiLoad 16/600 Superdex 200 column (GE Healthcare) (50 mM Tris pH 8, 150 mM NaCl, 0.5 mM DTT). The flow rate was set to 0.3 ml/min, and pure PARP1 containing fractions were concentrated (Amicon Ultra 4, 10 kDa MWCO) and stored at −80 °C.

### ATR-FTIR spectroscopy

Real-time ATR-FTIR spectroscopic measurements were performed as described previously^35,36^, with some adaptations. A Vertex 70 V spectrometer (Bruker) was equipped with a BioATR cell II (Bruker), which contained a multi-reflection silicon crystal. The penetration depth of the IR beam into the sample depends on the wavenumber, refractive indices, and angle of incidence, and is ~850 nm (calculated for 1000 cm^−1^, n_sample_ = 1.5, n_silicon_ = 3.4, and 45° angle of incidence). The spectral resolution was set to 4 cm^−1^, and for each spectrum 100 scans were performed. The temperature of the crystal was controlled via an external water bath and set to 20 °C. Unless stated otherwise, measurements were performed in Tris buffer (50 mM Tris pH 7.4, 150 mM NaCl).

Surface passivation of the ATR crystal: The modification of the crystal surface was performed as described^35,36^. Briefly, the surface was activated by treatment with H_2_SO_4_ and H_2_O_2_. Next, the crystal was heated to 50 °C and 20 mg/ml PEG–silane–biotin linker (5 kDa, Rapp Polymere) in 30 mM sodium acetate solution (pH 5.5) was added. After 30 min of incubation, the temperature was adjusted to 20 °C, and the biotin–PEG–silane linker solution was allowed to dry to achieve condensation of the silane groups. Then, the surface was washed thoroughly with Tris buffer and incubated in buffer for 1–2 h. This washing step was repeated and after 20 min of incubation in Tris buffer, the spectrum of the modified surface was set as background.

Immobilisation of biotinylated DNA strand break models: Ten to twenty picomole of annealed biotinylated DNA (DNA_blunt_, DNA_3’P_, DNA_5’P_ or DNA_nick_) were mixed with 10 pmol of streptavidin in 10 µl Tris buffer. The sample was applied to the biotinylated surface and incubated until the maximum signal was reached. This procedure was repeated until no further increase of signal was observed. The crystal was washed thoroughly with Tris buffer and the IR signal of immobilised DNA was set as background.

Analysis of DNA binding and PARylation of PARP1: First, 20 µl of 2 µM PARP1 in Tris buffer was added to the immobilised DNA. For technical reasons, measurements were started 5-10 s after addition of PARP1. Binding was followed for 20 min by taking spectra in time intervals of 1 min. Next, unbound PARP1 was removed by exchanging the buffer once. After 20 min of equilibration, a spectrum was recorded, which was used for structural analysis. To initiate the PARylation reaction by PARP1, 150 µl reaction buffer at the respective NAD^+^ concentration (0; 1; 10; 100; 500 µM) was added. Immediately after the addition, spectra were recorded for 80 min in 1-min time intervals. For technical reasons, the first recording of IR spectroscopic data started 1–2 s after addition of NAD^+^. After 80 min, loosely bound PARP1 was removed by washing thoroughly with NaCl buffer (50 mM Tris pH 7.4, 1 M NaCl). Finally, PARP1 was removed by washing thoroughly with SDS buffer (50 mM Tris pH 7.4, 150 mM NaCl, 1% SDS).

Analysis of inhibitor binding: First, 20 µl of 2 µM PARP1 in Tris buffer (50 mM Tris pH 7.8, 150 mM NaCl, 5 mM MgCl_2_) was added to the immobilised DNA_blunt_. After 20 min, a spectrum was recorded (designated ‘-Inhibitor’). Then, 15 µl of supernatant was removed, and 20 µl of inhibitor [veliparib or olaparib (both from Selleckchem)] was added to a final concentration of 10 µM. After 20 min, a spectrum was recorded (designated ‘+Inhibitor’). To analyse the inhibitor potency 50 µl NAD^+^ was added to a final concentration of 100 µM, and spectra were recorded for 30 min in 1-min time intervals. The impact of inhibitor on PARP1 binding to DNA was analysed by competitive binding, i.e., by adding free DNA_blunt_ to a final concentration of 2 µM to PARP1 bound to immobilised DNA_blunt_ in the presence or absence of 10 µM olaparib. Spectra were measured for 50 min in 2-min time intervals.

Spectral analysis: To analyse the binding of PARP1 to different DNA strand break models the IR spectrum of immobilised DNA was set as background. Binding kinetics were evaluated by tracking the intensity increase of the amide I band (1645 cm^−1^) over time. Data were fitted via the following mono-exponential function: $y = y_0 + (y_P - y_0) \cdot (1 - e^{ - kx})$; $y_0$ is the *y* intercept*,* i.e., the *y* value at which $x$ (time) is zero, $y_P$ ($y_{Plateau}$) is the *y* value reached at infinite times and *k* is the rate constant (min^−1^). It is important to note that the experimental approach limits the calculation of absolute values of rate constants, as the exact amount of immobilised DNA is an unknown parameter. Furthermore, due to the fast binding of PARP1 to the immobilised DNA, a time gap of some seconds between addition of PARP1 and the start of data acquisition cannot be avoided for technical reasons. Nevertheless, the method is highly suitable for a relative comparison of binding kinetics. Binding stoichiometries were evaluated by normalising the PARP1 signal to the signal of immobilised DNA. To do this, the intensity of the amide I band of PARP1 (1645 cm^−1^) was divided by the intensity of the band of the anti-symmetric phosphate vibration of DNA (1220 cm^−1^). To correct for different DNA lengths and varying numbers of phosphate groups, the values calculated were further multiplied by the respective number of phosphate groups. PARP1 dissociation upon NAD^+^ addition was analysed via the intensity of the amide II band (1548 cm^−1^) and fitted via the following mono-exponential function: $y = \left( {y_0 - y_P} \right)e^{ - kx} + y_P$. PAR formation upon NAD^+^ addition was analysed via the anti-symmetric phosphate vibration of PAR (1236 cm^−1^) and fitted via the following mono-exponential function: $y = y_0 + (y_P - y_0) \cdot (1 - e^{ - kx})$.

Reaction-induced difference spectroscopy of the amide I band: The amide I band (~1710–1595 cm^−1^) within the IR spectrum of proteins arises mainly from C=O stretching vibrations of the polypeptide backbone and is sensitive to protein secondary structures. Distinct frequencies within the amide I band can be assigned to distinct secondary structures, such as α-helices (1648–1657 cm^−1^), 3_10_-helices (1660–1666 cm^−1^), disordered structures (1642–1657 cm^−1^), β-sheets (1623–1641 and 1674–1695 cm^−1^), β-turns (1662–1686 cm^−1^) and extended β-sheets/amyloid aggregates (1630–1611 cm^−1^)^49,55^. Therefore, large proteins containing several secondary structures commonly display a single, broad amide I band, which is composed of overlapping amide I band components. Several techniques exist to resolve those structural components such as second-derivative analysis or Fourier self-deconvolution. Reaction-induced difference spectroscopy enables detection of subtle structural changes within proteins during protein reactions. After reaction induction (e.g. substrate addition), absorbance changes of proteins are monitored. Those absorbance changes are usually small compared to total absorbance. Therefore, difference spectra of two states of a protein are calculated. This eliminates all vibrational modes that do not change, and thereby resolves structural changes. Thus, remaining positive and negative bands within a difference spectrum represent structural components, which are characteristic for the respective state of a protein. To evaluate structural changes of PARP1 upon binding to different DNA strand break models or after addition of NAD^+^ or pharmacological PARP inhibitors, the respective amide I bands were first corrected for different water contents (O–H bending vibration overlays amide I band region). In a second step, a baseline correction between 1710 and 1595 cm^−1^ was performed. Then, amide I bands were normalised to the area under the curve and, finally, difference spectra were calculated.

### Gel-based assay for analysis of PARP1 automodification

PARP1 (0.5 µM) was incubated with DNA_blunt_, DNA_3’P_, DNA_5’P_ or DNA_nick_ (10 µM) for 15 min in Tris buffer (50 mM Tris pH 7.4, 150 mM NaCl) at room temperature. PARylation was started by the addition of a mixture of unlabelled and TAMRA-labelled NAD^+^ (20:1) to a final concentration of 100 µM^69^. PARylation was stopped by adding SDS loading dye and heating for 5 min at 95 °C. PARylation of PARP1 activated at different DNA strand break models was stopped after 5 min. For comparison of PARP1 and PARP1^E988K^ (activated at DNA_blunt_) PARylation was stopped after 1, 2, 5, 10 and 30 min. The extent of automodification was analysed via SDS-PAGE (4–20% precast polyacrylamide gel, Bio-Rad) and subsequent fluorescence detection.

### Gel-based assay for analysis of streptavidin PARylation

Streptavidin (2 µM) was pre-incubated with biotinylated or non-biotinylated DNA (2 µM) in Tris buffer (50 mM Tris pH 7.4, 150 mM NaCl) for 15 min at room temperature. Then, PARP1 (1 µM) was added, and binding of PARP1 to DNA was allowed for 20 min. Next, PARylation was started by addition of NAD^+^ to a final concentration of 500 µM. To exclude PARylation of DNA or an unspecific post-PARylation effect, streptavidin (2 µM) or biotinylated DNA (2 µM) were added after 60 min of PARylation as indicated, and samples were incubated for another 15 min at room temperature. Finally, PARylation was stopped by the addition of SDS loading dye and subsequent heating for 5 min at 95 °C. PARylation was analysed by SDS-PAGE followed by western blotting and immunodetection of PAR (10 H antibody^70^, purified from hybridoma cells, 1:250) and PARP1 (CII10 antibody^71^, purified from hybridoma cells, 1:250).

### Gel-based assay for analysis of DNA PARylation

PARP1 (1 µM) was incubated with Cy5-labelled DNA_3’P_ (0.1 µM) for 20 min at room temperature. The PARylation reaction was started by the addition of a mixture of unlabelled and TAMRA-labelled NAD^+^ ^69^ in ratio of 10:1 to a final concentration of 100 µM. After 30 min, PARylation was stopped by adding SDS to a final concentration of 1%. PARylation of DNA was analysed on a 20% sequencing gel followed by fluorescence detection.

### Enzymatic synthesis of ^13^C,^15^N-NAD^+^

Analysis of structural changes of PARP1 upon PARylation was challenging as two vibrational modes of PAR (1649 and 1605 cm^−1^) overlap the structure-sensitive amide I region (~1710–1595 cm^−1^). Therefore, isotopically labelled NAD^+^ was used. The enzymatic synthesis of isotopically labelled NAD^+^ was performed according to an adapted protocol from R. Martello (PhD thesis, University of Konstanz, 2013, http://nbn-resolving.de/urn:nbn:de:bsz:352-230252) and Balducci et al.^72^. ^13^C^15^N-ATP (Sigma-Aldrich) at a concentration of 1 mM was incubated for 1 h at RT together with 1 mM NMN (Sigma-Aldrich), 25 mU NMNAT1 (gift of M. Ziegler, University of Bergen, Norway) and 2.5 U inorganic pyrophosphatase from yeast (Roche) in 200 µl buffer (50 mM Tris pH 7.4; 150 mM NaCl; 10 mM MgCl_2_). Enzymes were removed by centrifugal filters [Nanosep, 10 kDa MWCO (Pall)]. To remove trace amounts of glycerol, which was used for storage of the filters but interferes with infrared spectroscopic analysis, centrifugal filters were first washed three times with 500 µl MilliQ before applying the enzymatic mixture. Conversion of ATP to NAD^+^ was analysed by HPLC equipped with a Hydro-RP 80 A column (250 × 4.6 mm, 4 micron) at a flow rate of 0.5 ml/min (solvent A: 8 mM ammonium acetate; solvent B: acetonitrile). The HPLC programme was set as follows: 0 min 1% B; 15 min 10% B; 30 min 15% B; 40 min 100% B; 50 min 100% B; 51 min 1% B; 65 min 1% B.

### Reporting summary

Further information on research design is available in the Nature Research Reporting Summary linked to this article.

## Supplementary information

Peer Review File

Supplementary Information

Reporting Summary

## Data Availability

Source data of Figs. 2b, c, d, 3c, d, 4b, c, 5a, d, 6a, b and Supplementary Figs. 1e, 2c, d, 3b, c, d, 4a, d, 5, 6b, c, d, f, 7a, b are provided as Source Data file. Further raw data and unprocessed data can be provided by the corresponding authors upon reasonable request.

## Source Data

Source Data

## References

[1] Rudolph J, Mahadevan J, Dyer P, Luger K (2018). Poly(ADP-ribose) polymerase 1 searches DNA via a ‘monkey bar’ mechanism. eLife.

[2] Ray Chaudhuri A, Nussenzweig A (2017). The multifaceted roles of PARP1 in DNA repair and chromatin remodelling. Nat. Rev. Mol. Cell Biol..

[3] Gibbs-Seymour I, Fontana P, Rack JGM, Ahel I (2016). HPF1/C4orf27 is a PARP-1-interacting protein that regulates PARP-1 ADP-ribosylation activity. Mol. Cell.

[4] Bonfiglio JJ (2017). Serine ADP-ribosylation depends on HPF1. Mol. Cell.

[5] Jungmichel S (2013). Proteome-wide identification of Poly(ADP-Ribosyl)ation targets in different genotoxic stress responses. Mol. Cell.

[6] Zhang Y, Wang J, Ding M, Yu Y (2013). Site-specific characterization of the Asp- and Glu-ADP-ribosylated proteome. Nat. Methods.

[7] Gibson BA (2016). Chemical genetic discovery of PARP targets reveals a role for PARP-1 in transcription elongation. Science.

[8] Langelier M-F, Riccio AA, Pascal JM (2014). PARP-2 and PARP-3 are selectively activated by 5′ phosphorylated DNA breaks through an allosteric regulatory mechanism shared with PARP-1. Nucleic Acids Res..

[9] Murai J (2012). Trapping of PARP1 and PARP2 by clinical PARP inhibitors. Cancer Res..

[10] Satoh MS, Lindahl T (1992). Role of poly(ADP-ribose) formation in DNA repair. Nature.

[11] Steffen J. D., McCauley M. M. & Pascal J. M. Fluorescent sensors of PARP-1 structural dynamics and allosteric regulation in response to DNA damage. *Nucleic Acids Res.* **20**, 9771–9783 (2016). 10.1093/nar/gkw710 PMC5175350 27530425

[12] Chambon P, Weill JD, Mandel P (1963). Nicotinamide mononucleotide activation of a new DNA-dependent polyadenylic acid synthesizing nuclear enzyme. Biochem. Biophys. Res. Commun..

[13] Langelier MF, Planck JL, Roy S, Pascal JM (2011). Crystal structures of poly(ADP-ribose) polymerase-1 (PARP-1) zinc fingers bound to DNA: structural and functional insights into DNA-dependent PARP-1 activity. J. Biol. Chem..

[14] Eustermann S (2015). Structural basis of detection and signaling of DNA single-strand breaks by human PARP-1. Mol. Cell.

[15] Lonskaya I (2005). Regulation of poly(ADP-ribose) polymerase-1 by DNA structure-specific binding. J. Biol. Chem..

[16] D’Silva I (1999). Relative affinities of poly(ADP-ribose) polymerase and DNA-dependent protein kinase for DNA strand interruptions. Biochimica et. biophysica acta.

[17] Kun E, Kirsten E, Mendeleyev J, Ordahl CP (2004). Regulation of the enzymatic catalysis of Poly(ADP-ribose) polymerase by dsDNA, polyamines, Mg2+, Ca2+, Histones H1 and H3, and ATP. Biochemistry.

[18] Ali AAE (2012). The zinc-finger domains of PARP1 cooperate to recognize DNA strand breaks. Nat. Struct. Amp; Mol. Biol..

[19] Eustermann S (2011). The DNA-binding domain of human PARP-1 interacts with DNA single-strand breaks as a monomer through its second zinc finger. J. Mol. Biol..

[20] Karlberg T, Langelier M-F, Pascal JM, Schüler H (2013). Structural biology of the writers, readers, and erasers in mono- and poly(ADP-ribose) mediated signaling. Mol. Asp. Med..

[21] Langelier M-F, Ruhl DD, Planck JL, Kraus WL, Pascal JM (2010). The Zn3 domain of human Poly(ADP-ribose) polymerase-1 (PARP-1) functions in both DNA-dependent Poly(ADP-ribose) synthesis activity and chromatin compaction. J. Biol. Chem..

[22] Langelier M-F, Planck JL, Roy S, Pascal JM (2012). Structural basis for DNA damage–dependent Poly(ADP-ribosyl)ation by Human PARP-1. Science.

[23] Loeffler PA (2011). Structural studies of the PARP-1 BRCT domain. BMC Struct. Biol..

[24] Barkauskaite E, Jankevicius G, Ahel I (2015). Structures and mechanisms of enzymes employed in the synthesis and degradation of PARP-dependent protein ADP-ribosylation. Mol. Cell.

[25] Steffen JD, Brody JR, Armen RS, Pascal JM (2013). Structural implications for selective targeting of PARPs. Front. Oncol..

[26] Langelier M-F, Pascal JM (2013). PARP-1 mechanism for coupling DNA damage detection to poly(ADP-ribose) synthesis. Curr. Opin. Struct. Biol..

[27] Langelier MF, Zandarashvili L, Aguiar PM, Black BE, Pascal JM (2018). NAD(+) analog reveals PARP-1 substrate-blocking mechanism and allosteric communication from catalytic center to DNA-binding domains. Nat. Commun..

[28] Dawicki-McKenna Jennine M (2015). PARP-1 activation requires local unfolding of an autoinhibitory domain. Mol. Cell.

[29] Mendoza-Alvarez H, Alvarez-Gonzalez R (1993). Poly(ADP-ribose) polymerase is a catalytic dimer and the automodification reaction is intermolecular. J. Biol. Chem..

[30] Pion E (2005). DNA-induced dimerization of poly(ADP-ribose) polymerase-1 triggers its activation. Biochemistry.

[31] Lilyestrom W, van der Woerd MJ, Clark N, Luger K (2010). Structural and biophysical studies of human PARP-1 in complex with damaged DNA. J. Mol. Biol..

[32] Liu L (2017). PARP1 changes from three-dimensional DNA damage searching to one-dimensional diffusion after auto-PARylation or in the presence of APE1. Nucleic Acids Res..

[33] Goormaghtigh E, Raussens V, Ruysschaert JM (1999). Attenuated total reflection infrared spectroscopy of proteins and lipids in biological membranes. Biochimica et. biophysica acta.

[34] Nyquist RM, Ataka K, Heberle J (2004). The molecular mechanism of membrane proteins probed by evanescent infrared waves. Chembiochem.

[35] Krüger A, Stier A, Fischbach A, Bürkle A, Hauser K, Mangerich A (2019). Interactions of p53 with poly(ADP-ribose) and DNA induce distinct changes in protein structure as revealed by ATR-FTIR spectroscopy. Nucleic Acids Res..

[36] Krüger A, Bürkle A, Mangerich A, Hauser K (2018). A combined approach of surface passivation and specific immobilization to study biomolecules by ATR-FTIR spectroscopy. Biomed. Spectrosc. Imaging.

[37] Sukhanova MV (2016). Single molecule detection of PARP1 and PARP2 interaction with DNA strand breaks and their poly(ADP-ribosyl)ation using high-resolution AFM imaging. Nucleic Acids Res..

[38] Banyay M, Sarkar M, Gräslund A (2003). A library of IR bands of nucleic acids in solution. Biophysical Chem..

[39] Langelier MF, Riccio AA, Pascal JM (2014). PARP-2 and PARP-3 are selectively activated by 5’ phosphorylated DNA breaks through an allosteric regulatory mechanism shared with PARP-1. Nucleic Acids Res..

[40] Langelier M-F, Eisemann T, Riccio AA, Pascal JM (2018). PARP family enzymes: regulation and catalysis of the poly(ADP-ribose) posttranslational modification. Curr. Opin. Struct. Biol..

[41] Pascal JM (2018). The comings and goings of PARP-1 in response to DNA damage. DNA Repair.

[42] Bayer EA, Ehrlich-Rogozinski S, Wilchek M (1996). Sodium dodecyl sulfate-polyacrylamide gel electrophoretic method for assessing the quaternary state and comparative thermostability of avidin and streptavidin. Electrophoresis.

[43] Waner MJ, Navrotskaya I, Bain A, Oldham ED, Mascotti DP (2004). Thermal and sodium dodecylsulfate induced transitions of streptavidin. Biophysical J..

[44] Talhaoui I (2016). Poly(ADP-ribose) polymerases covalently modify strand break termini in DNA fragments in vitro. Nucleic Acids Res..

[45] Munnur D, Ahel I (2017). Reversible mono-ADP-ribosylation of DNA breaks. FEBS J..

[46] Belousova EA, Ishchenko АA, Lavrik OI (2018). Dna is a new target of Parp3. Sci. Rep..

[47] Zarkovic G (2018). Characterization of DNA ADP-ribosyltransferase activities of PARP2 and PARP3: new insights into DNA ADP-ribosylation. Nucleic Acids Res..

[48] Fischbach A (2018). The C-terminal domain of p53 orchestrates the interplay between non-covalent and covalent poly(ADP-ribosyl)ation of p53 by PARP1. Nucleic Acids Res..

[49] Barth A (2007). Infrared spectroscopy of proteins. Biochimica et. Biophysica Acta.

[50] Alemasova EE, Lavrik OI (2019). Poly(ADP-ribosyl)ation by PARP1: reaction mechanism and regulatory proteins. Nucleic Acids Res..

[51] Marsischky GT, Wilson BA, Collier RJ (1995). Role of glutamic acid 988 of human poly-ADP-ribose polymerase in polymer formation. Evidence for active site similarities to the ADP-ribosylating toxins. J. Biol. Chem..

[52] Rolli V, O’Farrell M, Menissier-de Murcia J, de Murcia G (1997). Random mutagenesis of the poly(ADP-ribose) polymerase catalytic domain reveals amino acids involved in polymer branching. Biochemistry.

[53] Beneke S, Scherr AL, Ponath V, Popp O, Burkle A (2010). Enzyme characteristics of recombinant poly(ADP-ribose) polymerases-1 of rat and human origin mirror the correlation between cellular poly(ADP-ribosyl)ation capacity and species-specific life span. Mechanisms Ageing Dev..

[54] Rank L (2016). Analyzing structure-function relationships of artificial and cancer-associated PARP1 variants by reconstituting TALEN-generated HeLa PARP1 knock-out cells. Nucleic Acids Res..

[55] Yang H, Yang S, Kong J, Dong A, Yu S (2015). Obtaining information about protein secondary structures in aqueous solution using Fourier transform IR spectroscopy. Nat. Protoc..

[56] Chirgadze YN, Nevskaya NA (1976). Infrared spectra and resonance interaction of amide-I vibration of the paraellel-chain pleated sheets. Biopolymers.

[57] Krimm S, Bandekar J (1986). Vibrational spectroscopy and conformation of peptides, polypeptides, and proteins. Adv. Protein Chem..

[58] Pommier Y, O’Connor MJ, de Bono J (2016). Laying a trap to kill cancer cells: PARP inhibitors and their mechanisms of action. Sci. Transl. Med..

[59] Shen Y, Aoyagi-Scharber M, Wang B (2015). Trapping Poly(ADP-Ribose) polymerase. J. Pharmacol. Exp. therapeutics.

[60] Hopkins TA (2015). Mechanistic dissection of PARP1 trapping and the impact on in vivo tolerability and efficacy of PARP inhibitors. Mol. Cancer Res..

[61] Wei H, Yu X (2016). Functions of PARylation in DNA damage repair pathways. Genomics, Proteom. Bioinforma..

[62] Liu C, Vyas A, Kassab MA, Singh AK, Yu X (2017). The role of poly ADP-ribosylation in the first wave of DNA damage response. Nucleic Acids Res..

[63] Weinfeld M, Mani RS, Abdou I, Aceytuno RD, Glover JNM (2011). Tidying up loose ends: the role of polynucleotide kinase/phosphatase in DNA strand break repair. Trends Biochem. Sci..

[64] Menissier de Murcia J (2003). Functional interaction between PARP-1 and PARP-2 in chromosome stability and embryonic development in mouse. EMBO J..

[65] Gagne JP (2008). Proteome-wide identification of poly(ADP-ribose) binding proteins and poly(ADP-ribose)-associated protein complexes. Nucleic Acids Res..

[66] Kurzban GP, Bayer EA, Wilchek M, Horowitz PM (1991). The quaternary structure of streptavidin in urea. J. Biol. Chem..

[67] Murai J (2014). Stereospecific PARP trapping by BMN 673 and comparison with olaparib and rucaparib. Mol. Cancer Therapeut..

[68] Langelier MF, Planck JL, Servent KM, Pascal JM (2011). Purification of human PARP-1 and PARP-1 domains from *Escherichia coli* for structural and biochemical analysis. Methods Mol. Biol..

[69] Wang Y, Rosner D, Grzywa M, Marx A (2014). Chain-terminating and clickable NAD(+) analogues for labeling the target proteins of ADP-ribosyltransferases. Angew. Chem..

[70] Kawamitsu H (1984). Monoclonal antibodies to poly(adenosine diphosphate ribose) recognize different structures. Biochemistry.

[71] Lamarre D (1988). Structural and functional analysis of poly(ADP ribose) polymerase: an immunological study. Biochimica et. biophysica acta.

[72] Balducci E (1995). Assay methods for nicotinamide mononucleotide adenylyltransferase of wide applicability. Anal. Biochem..

