# Supplementary material for: Real-time monitoring of PARP1-dependent PARylation by ATR-FTIR spectroscopy
Source: Nat Commun. 2020 May 1;11:2174. doi: 10.1038/s41467-020-15858-w (PMC7195430; doi:10.1038/s41467-020-15858-w)
Supplement: Supplementary file 2 — Supplementary Information [file 41467_2020_15858_MOESM2_ESM.pdf]

SUPPLEMENTARY INFORMATION

Real-time monitoring of PARP1-dependent PARylation by ATR-FTIR-spectroscopy

Annika Krüger[1,2], Alexander Bürkle[1], Karin Hauser[2,*] and Aswin Mangerich[1,*]

[1] Department of Biology, University of Konstanz, Konstanz 78464, Germany
[2] Department of Chemistry, University of Konstanz, Konstanz 78464, Germany

* To whom correspondence should be addressed:

Aswin Mangerich, Tel: +49 7531 88-4067, Fax: +49 7531 88-4033, email: aswin.mangerich@uni-konstanz.de;
Karin Hauser, Tel: +49 7531 88-5356, Fax: +49 7531 88-3139, email: karin.hauser@uni-konstanz.de;

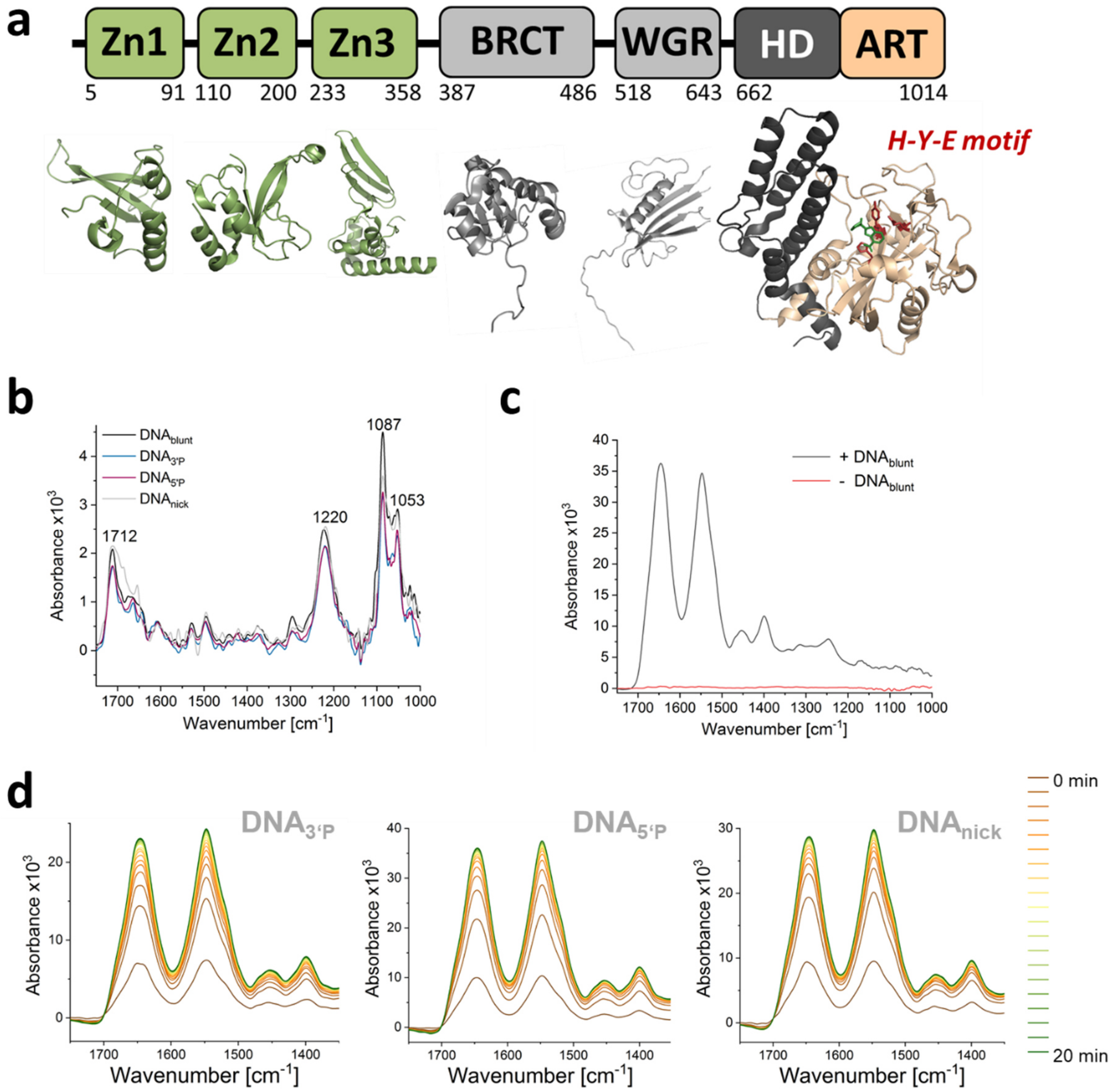

Supplementary Fig. 1. a Domain organization of PARP1. PARP1 contains three zinc-finger domains Zn1 (pdb: 3oda), Zn2 (pdb: 3ode) and Zn3 (pdb: 2jvn), a BRCT domain (pdb: 2le0), a WGR domain (pdb: 2cr9) and a catalytic domain harbouring an autoinhibitory HD domain and the catalytically active ART domain (pdb: 3gn7). The catalytic domain is in complex with veliparib. The catalytic conserved H-Y-E motif is highlighted. **b** Representative spectra of immobilized DNA_{blunt}, $DNA_{3'P}$, $DNA_{5'P}$ and DNA_{nick}. Spectra of streptavidin were subtracted, respectively. **c** Comparison of PARP1 binding to DNA_{blunt}, which was immobilized via streptavidin (+DNA_{blunt}), or streptavidin alone, i.e., without DNA_{blunt} (-DNA_{blunt}). **d** Representative time-dependent spectra of PARP1 binding to immobilized $DNA_{3'P}$, $DNA_{5'P}$ and DNA_{nick}. Source data are provided as a Source Data file.

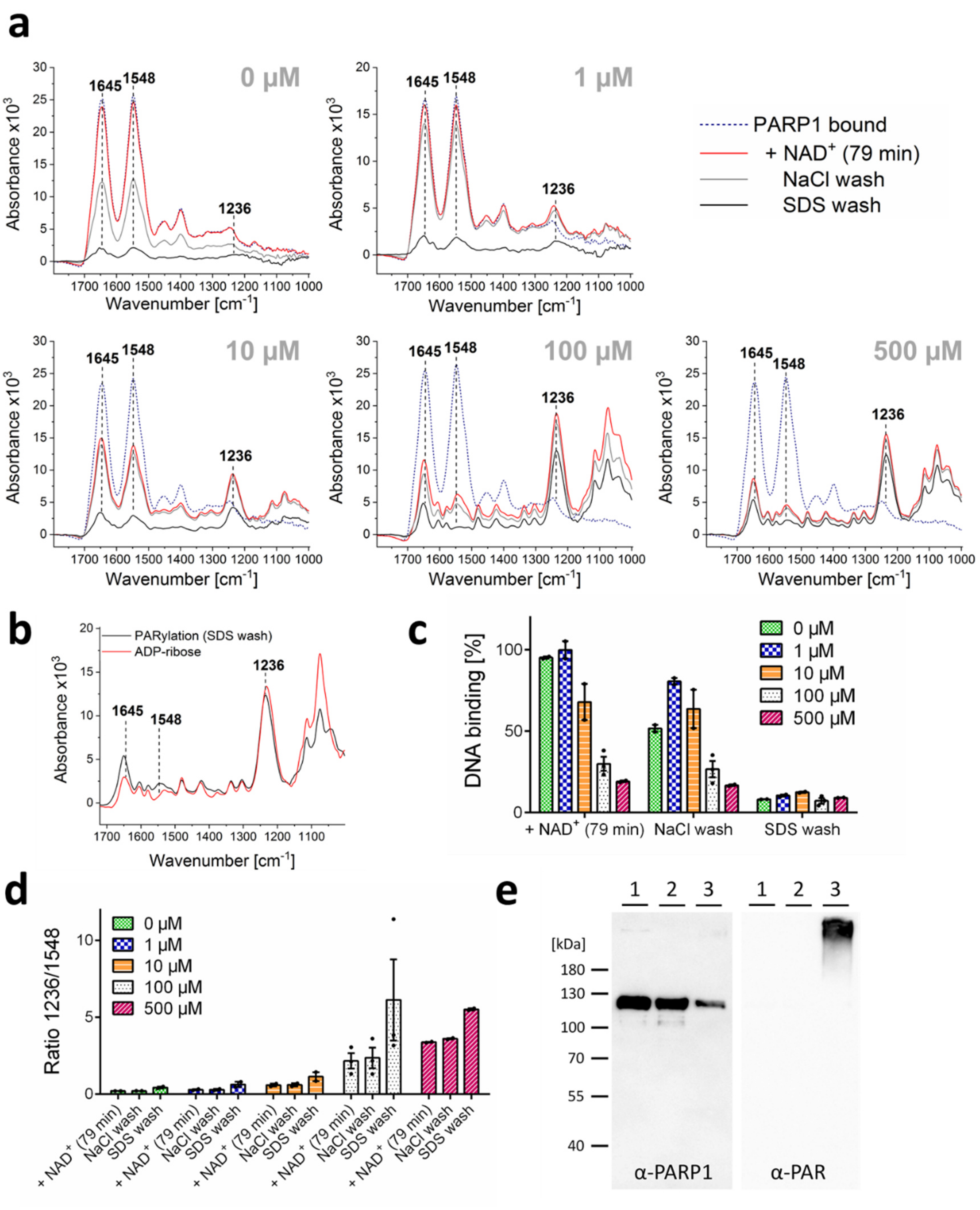

Supplementary Fig. 2. a Representative spectra of PARP1 bound to DNA$_{blunt}$ before ('PARP1 bound') and after the addition of various concentrations (0-500 µM) of NAD$^+$ ('79 min') and subsequent washing with 1 M NaCl ('NaCl wash') and 1% SDS ('SDS wash'). **b-d** Evaluation of **a**. **b** Comparison of the spectrum of **a**, bottom right panel (addition of 500 µM NAD$^+$, 'SDS wash'), with the spectrum of ADP-ribose (Fig. 3a). **c** Quantification of PARP1 binding to DNA$_{blunt}$. Intensities of amide II bands (1548 cm^{-1}) were analysed. 'PARP1 bound' was set to 100%. **d** Analysis of 'trans-PARylation'. Ratio of anti-symmetric phosphate vibration of PAR (1236 cm^{-1}) and amide II band of PARP1 (1548 cm^{-1}) was calculated. **c, d** Means ± SEM of n=3 (100 µM) and n=2 (0, 1, 10 and 500 µM) independent experiments respectively. **e** Analysis of the supernatant from an ATR-FTIR spectroscopic experiment by immunoblotting. PARP1 was detected via CII10 antibody and PAR was detected via 10H antibody. Lanes 1+2: Analysis of the efficiency of PARP1 binding to immobilized DNA$_{blunt}$ [before (1) and after (2) addition of PARP1 to immobilized DNA$_{blunt}$]. Lane 3: Analysis of the extent of auto-modification of PARP1 after NAD$^+$ addition (79 min). Source data are provided as a Source Data file.

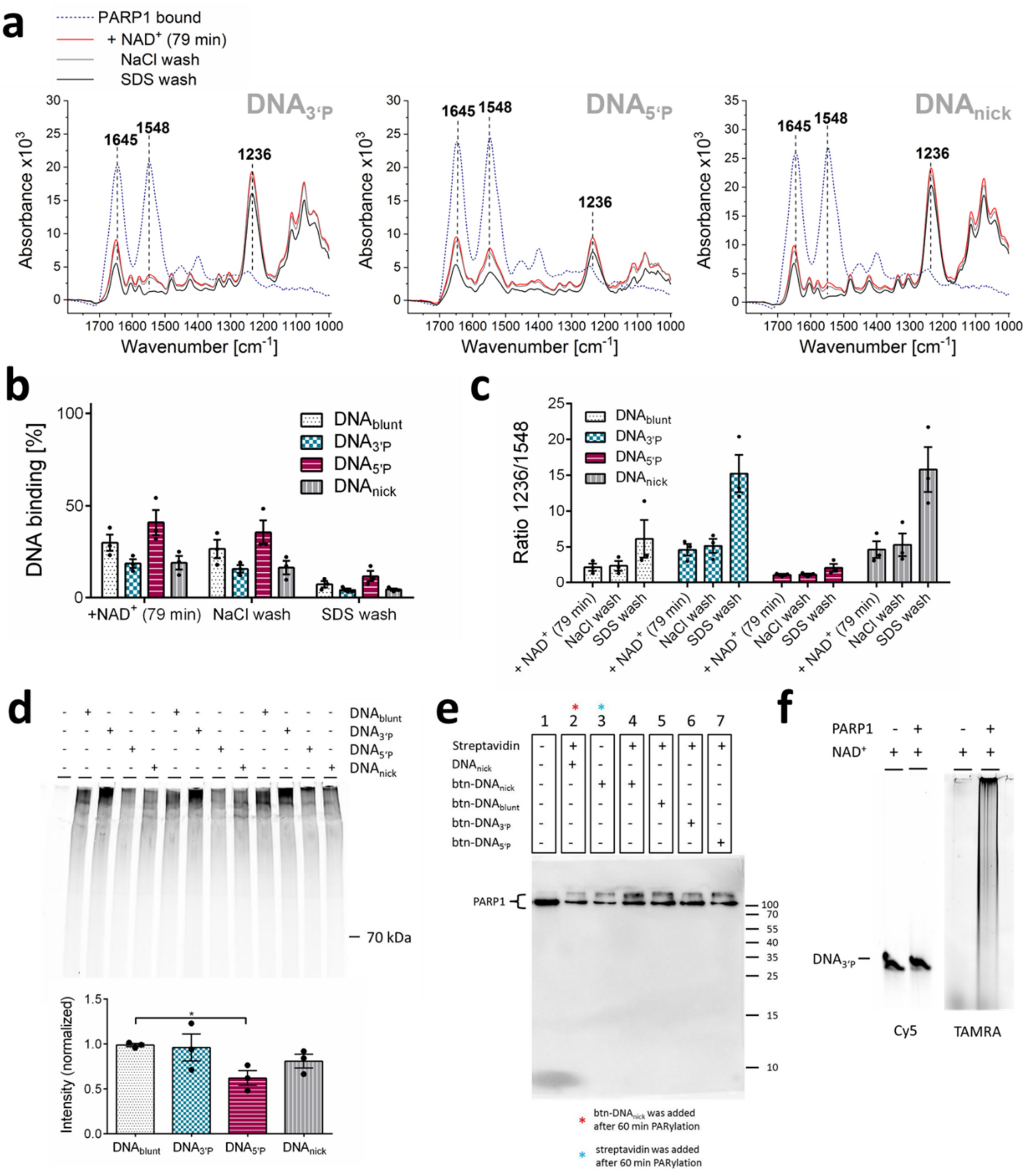

Supplementary Fig. 3. a Representative spectra of PARP1 bound to $DNA_{3'P}$, $DNA_{5'P}$ or DNA_{nick} before ('PARP1 bound') and after the addition of 100 µM of NAD^+ ('79 min') and subsequent washing with 1 M NaCl ('NaCl wash') and 1% SDS ('SDS wash'). **b,c** Evaluation of **a** and Supplementary Fig. 2a (100 µM NAD^+). **b** Quantification of PARP1 binding to DNA_{blunt}, $DNA_{3'P}$, $DNA_{5'P}$ or DNA_{nick}. Intensities of amide II bands (1548 cm^{-1}) were analysed. 'PARP1 bound' was set to 100%. **c** Analysis of 'trans-PARylation'. Ratio of anti-symmetric phosphate vibration of PAR (1236 cm^{-1}) and amide II band of PARP1 (1548 cm^{-1}) was calculated. **b,c** Means ± SEM of n=3 independent experiments. **d** Analysis of auto-modification of PARP1 via a gel-based assay using fluorescently labelled NAD^+. PARylation was started by the addition of 100 µM TAMRA-labelled NAD^+. Upper panel: Representative gel with three technical replicates. Lower panel: Densitometric quantification of signal intensities. Means ± SEM of n=3 independent experiments. '*' indicates $p<0.05$, statistical analysis was performed using an unpaired two-sided t-test. **e** Immunodetection of PARP1 corresponding to Fig. 4d. **f** Analysis of covalent PARylation of $DNA_{3'P}$ via a gel-based assay detecting Cy5-labelled $DNA_{3'P}$ ('Cy5') or TAMRA-labelled NAD^+ ('TAMRA'). Source data are provided as a Source Data file.

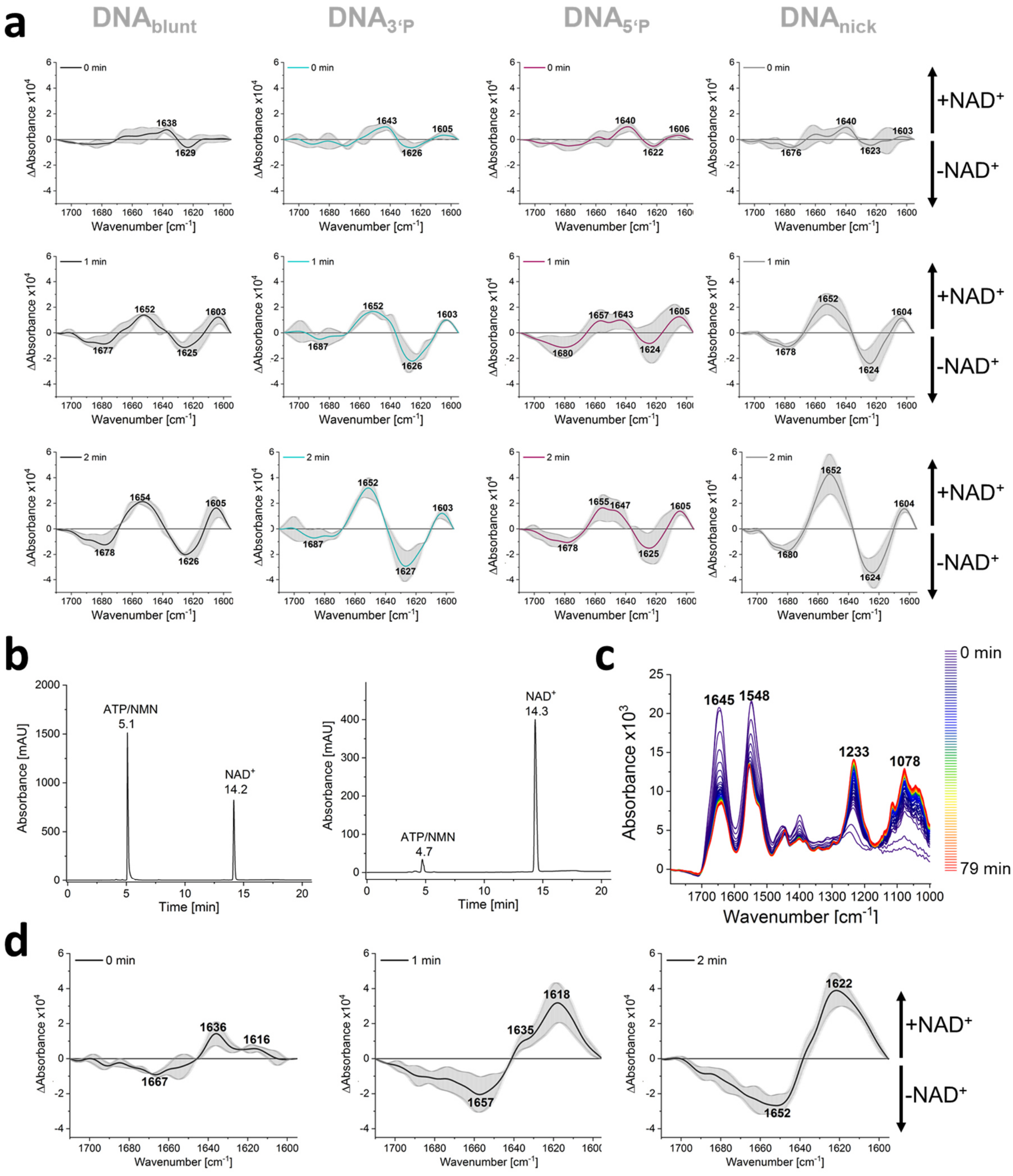

Supplementary Fig. 4. a Individual average curves including SDs (grey), which correspond to data shown in Fig. 5a. **b** Analysis of enzymatic synthesis of ^{13}C,^{15}N-NAD^+ via HPLC. ^{13}C,^{15}N-ATP and NMN were incubated with NMNAT1 in the absence (*left panel*) or in the presence (*right panel*) of PPase. **c** Representative time-dependent spectra following the addition of 100 µM ^{13}C,^{15}N-NAD^+ to PARP1 bound to immobilized DNA_{blunt}. Amide I (1645 cm^{-1}) and amide II (1548 cm^{-1}) bands of PARP1 and anti-symmetric (1233 cm^{-1}) and symmetric (1078 cm^{-1}) phosphate vibrations of generated isotopically labelled PAR are indicated. **d** Average curves and SDs (grey), which correspond to data shown in Fig. 5d. Source data are provided as a Source Data file.

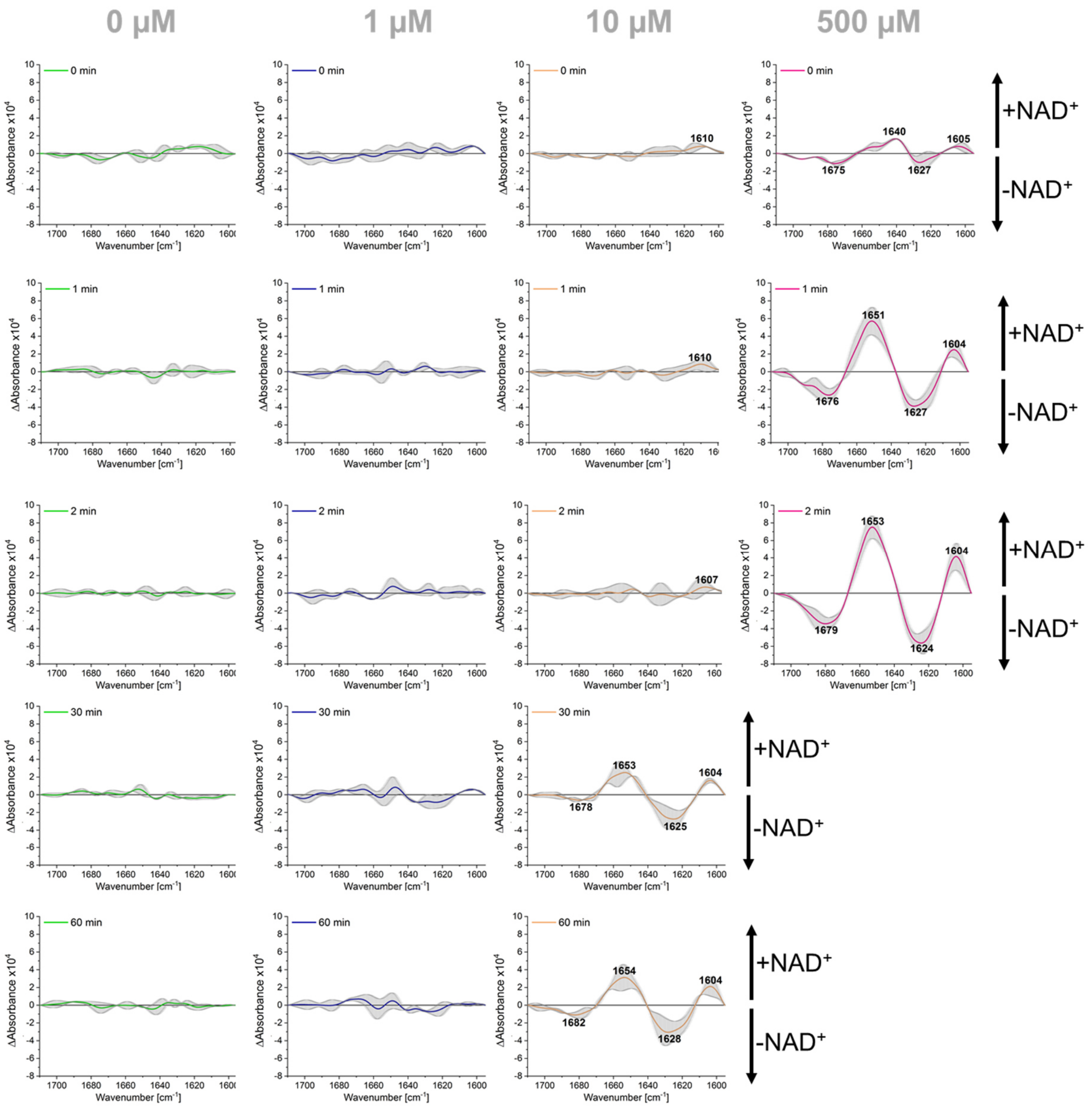

Supplementary Fig. 5. Secondary structure analysis of PARP1 upon addition of various NAD^+ concentrations. Difference spectra of amide I bands of PARP1 (1710-1595 cm^{-1}) before and after the addition of NAD^+ (0, 1, 2, 30 and 60 min) were calculated. Average curves and SDs (grey) of n=2 independent experiments are plotted, respectively. Source data are provided as a Source Data file.

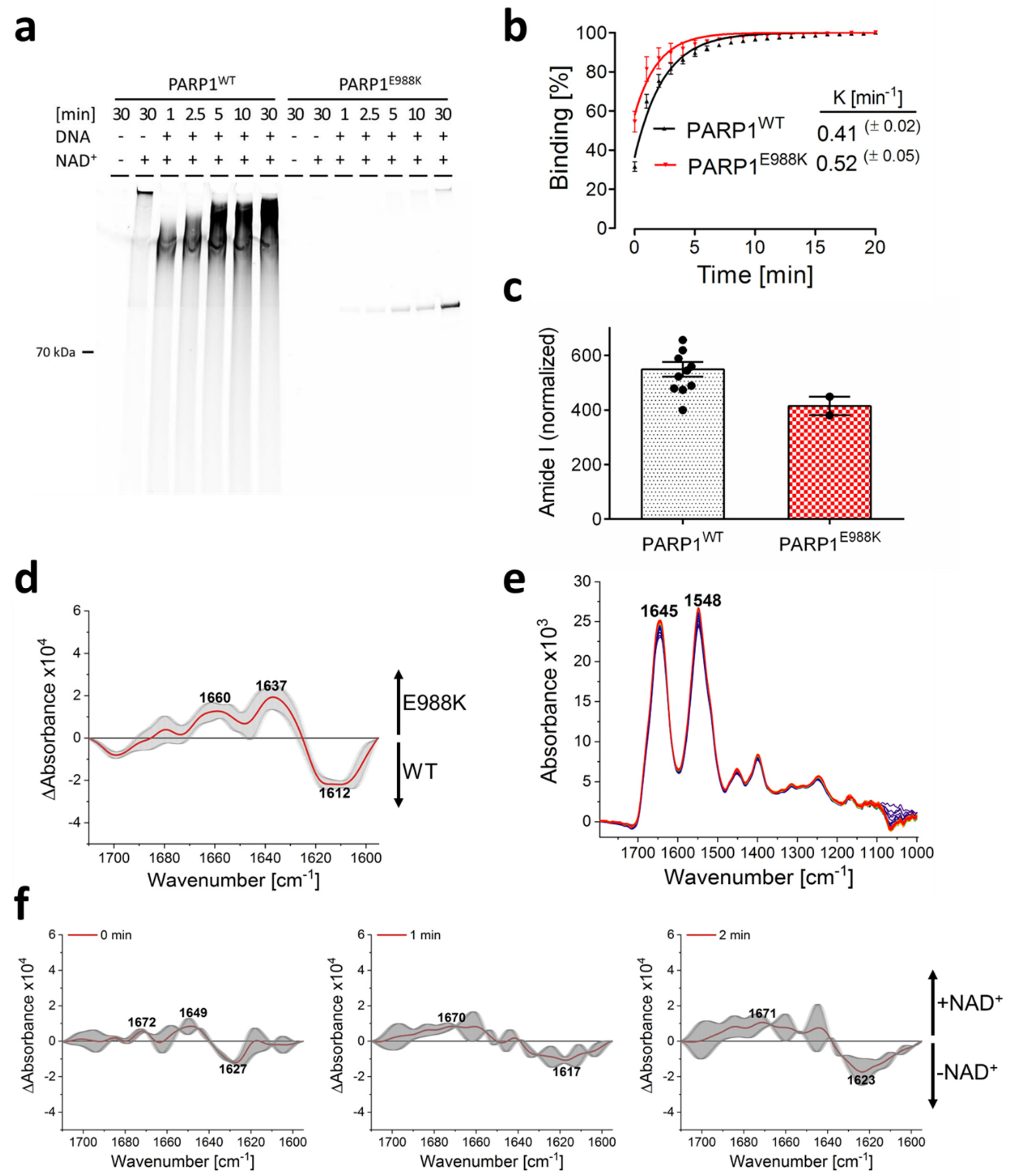

Supplementary Fig. 6. Analysis of PARP1 variant E988K. **a** Comparison of auto-modification of $PARP1^{WT}$ and $PARP1^{E988K}$ via a gel-based assay using fluorescently labelled NAD^+. PARylation was started by the addition of 100 µM TAMRA-labelled NAD^+. **b** Evaluation of time-dependent binding of $PARP1^{WT}$ and $PARP1^{E988K}$ to DNA_{blunt}. '0 min' refers to start of measurements. Signal intensities of amide I bands (1645 cm^{-1}) at 20 min were set to 100%. Binding kinetics were calculated via a mono-exponential fit function. $PARP1^{E988K}$: Means ± SEM of n=2 independent experiments. Data of $PARP1^{WT}$ was taken from Fig. 2. **c** Comparison of the amount of $PARP1^{WT}$ and $PARP1^{E988K}$ bound to DNA_{blunt} after 20 min of incubation (data from same experiments as in b). Amide I bands (1645 cm^{-1}) were normalized to the amount of immobilized DNA (1220 cm^{-1}). Data of $PARP1^{WT}$ was taken from Fig. 2. **d** Secondary structure analysis. Difference spectra of amide I bands of $PARP1^{WT}$ and $PARP1^{E988K}$ bound to DNA_{blunt} were calculated. Average curve and SD (grey) of 6 difference spectra are plotted ($PARP1^{WT}$: n=3 independent experiments; $PARP1^{E988K}$: n=2 independent experiments). **e** Representative time-resolved spectra subsequent to the addition of 100 µM NAD^+ to $PARP1^{E988K}$ bound to immobilized DNA_{blunt}. Amide I (1645 cm^{-1}) and amide II (1548 cm^{-1}) bands are indicated. **f** Secondary structure analysis of $PARP1^{E988K}$ upon addition of 100 µM NAD^+. Difference spectra of amide I bands (1710-1595 cm^{-1}) before and after the addition of NAD^+ (0, 1 and 2 min) were calculated. Average curves and SDs (grey) of n=2 independent experiments. Source data are provided as a Source Data file.

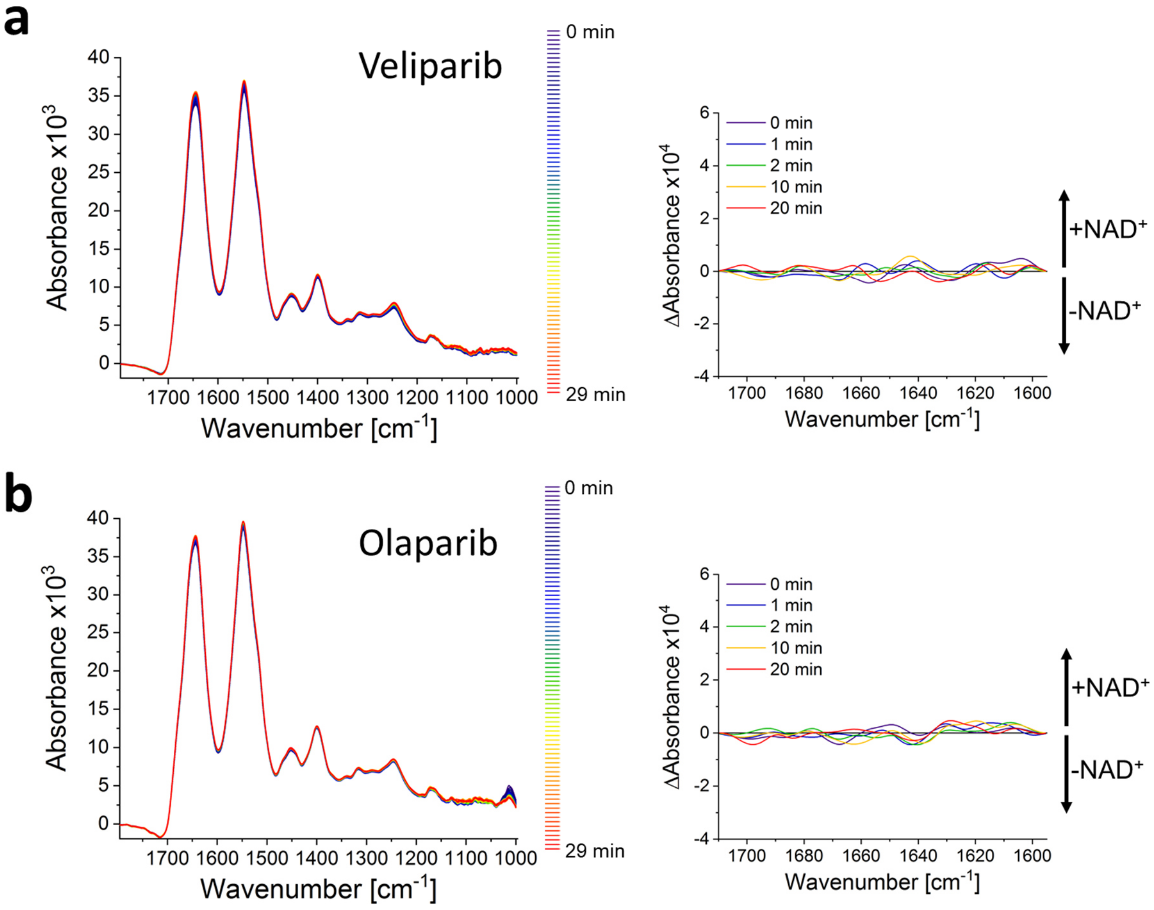

Supplementary Fig. 7. a Addition of 100 µM NAD^+ to PARP1 bound to immobilized DNA_{blunt} after preincubation with veliparib **a** or olaparib **b**. *Left panels*: Time-dependent IR spectra. *Right panels*: Secondary structure analysis of PARP1. Difference spectra of amide I bands (1710-1595 cm^{-1}) before and after the addition of NAD^+ (0, 1, 2, 10 and 20 min) were calculated and do not indicate significant structural changes. These control experiments show that structural changes of PARP1 are specifically observed after binding of NAD^+ to the catalytic centre. Source data are provided as a Source Data file.
