# Supplementary material for: Real-time monitoring of PARP1-dependent PARylation by ATR-FTIR spectroscopy
Source: Nat Commun. 2020 May 1;11:2174. doi: 10.1038/s41467-020-15858-w (PMC7195430; doi:10.1038/s41467-020-15858-w)
Supplement: Supplementary file 1 — Peer Review File [file 41467_2020_15858_MOESM1_ESM.pdf]

Reviewers' comments:

Reviewer #1 (Remarks to the Author):

Manuscript 223061
Krüger et al. Real-time monitoring of PARP1-dependent PARylation by ATR-FTIR-spectroscopy

Krüger et al use the spectroscopic technique ATR-FTIR to report on the kinetics of PARP-1 (i) binding to DNA, (ii) catalyzing poly(ADP-ribose) formation, and (iii) releasing from DNA. They also investigate the influence of PARP inhibitors on this process. The study addresses interesting aspects of PARP-1 function, probably most importantly the complicated process of PARP-1 release from DNA due to poly(ADP-ribose) catalysis. While the focus of the study is interesting and the ATR-FTIR approach is novel, it is difficult to draw confident conclusions from the results. The study lacks some controls that could test whether the interpretation of the results is correct. Some of the difference spectra are stated as having non-significant features (Fig. 2D), but it is not clear why they are deemed to be non-significant. Some controls to demonstrate the power and the sensitivity of the technique are warranted. One way to address these issues would be to use mutant versions of PARP-1 that have known characteristics in terms of binding affinity or ability to be activated or ability to bind NAD+, and to demonstrate that these mutants introduce the expected changes in the ATR-FITR spectra. As the study currently stands, it is not entirely clear that the observations have been interpreted correctly. This concern applies mainly to the interpretation of the ~1640 cm-1 band as a structural change in PARP-1. Another issue concerns the setup of the experiment. It appears that the majority of the poly(ADP-ribose) is attached to either the DNA or the streptavidin, so not the major target of PARP-1 modification. Thus, the build up of poly(ADP-ribose) on PARP-1 itself is not really being measured, and is instead inferred to exist. Would other attachment strategies (i.e. neutravidin, longer DNAs) potentially overcome this issue.

Other concerns:

There are no error bars on Figure S3e, suggesting this experiment was only performed once. Since there are some conclusions drawn in comparing these data between the different DNAs used, the experiment should be done multiple times to provide SEM and a sense of the variability.

The use of the term "allosteric binding" is confusing in some places:

"...caused by allosteric binding of NAD+ to the catalytic centre.."

"The allosteric binding of NAD+..."

It seems odd to refer to NAD+ binding as allosteric, unless talking specifically about the effect on DNA binding.

Can it be directly tested whether the DNA and/or the streptavidin are actually modified with poly(ADP-ribose)?

Figure 4: Legend says D, but there is no panel labelled as D.

"Notably, as we observed strong automodification of PARP1 in the gel-based assay, this suggests that automodification of PARP1 mainly occurs, after initial activation, in the absence of DNA.”

Since the poly(ADP-ribose) produced in the ATR-FITR setup is not really directed at PARP-1, whereas it is directed toward PARP-1 in the gel-based assay, it does not seem safe to suggest the statement above.

Reviewer #2 (Remarks to the Author):

The authors use ATR-FTIR to study the binding, activity (PARylation), and release of PARP1 from various forms of DNA damage. Overall the manuscript is very well written, and it is clear that the experiments were performed with great care and attention to detail. While the novelty of the findings presented in this manuscript is not overwhelming, confirmation of previous observations by this orthogonal and novel approach are a valuable contribution to the literature. I recommend acceptance upon minor modifications. See more detailed comments below:

Fig. 2B: Should the percent binding not start at 0% in the fitting procedure? It appears to start at ~30%, and it seems that a fit through 0,0 would lead to non-first-order kinetics, which would be problematic for the interpretations made in the text. Additionally, under the conditions of 2 μM PARP1 and with an apparent rate of ~0.5/min, one can calculate on-rates of ~4 x 103 M-1s-1. These values are very slow compared to the rates of association of most DNA-binding proteins (>>106 M-1s-1), in particular the measured value for PARP1 of 109 M-1s-1 (see PMID 30088474). Does the apparent slow on-rate have something to do with the surface chemistry of the experimental system? The authors need to address these issues of data-fitting, interpretation, and comparison to existing literature.

Fig. 3B,C: The authors do a nice job of discriminating PAR formation and dissociation of PARP1, and their washing experiments nicely demonstrate that PARylation of non-self (DNA and/or streptavidin) must be occurring. As no one has previously demonstrated PARylation of streptavidin, a simple TAMRA-labeled NAD+ - based assay using soluble PARP1 and soluble streptavidin would be a good supplementary figure to include here.

Fig. 3D: My back-of-the-envelope calculations (assuming 10 pmol of DNA, each bound to 1 PARP1 with a turnover number of ~5 s-1) suggests that the amount of NAD+ consumption is about as expected from solution-based measurements, which is reassuring and suggests that PARP1 bound to the surface is intact and catalytically active. A comparison to the existing literature re: turnover numbers could be made here and would strengthen the authors’ conclusions.

Fig. 4: Although the differences in rates of PARylation and PARP1 release are relatively minor, the correlation between automodification and release is as expected, thus making these data believable. Given that the differences between the different forms of DNA damage are less than 2-fold, the interpretations presented in the Discussion appear too strong.

Fig. 5: The care with which the authors perform the analyses of changes in the conformation of PARP1 upon PARylation are commendable. That is, using the “trick” of isotopically labeled NAD+ in order to better resolve the signal of interest is excellent. Also, it is evident that they have carefully considered concentrations of NAD+ and extents of reaction in their sample preparation. The data are convincing but shed little insight into the conformational changes that occur upon binding of NAD+. The finding of a slight increase in some disordered protein or more beta-sheets, although correlating with Pascal’s allosteric-mechanism (reference #26), does not actually identify which part of the protein is undergoing this conformational change. Given that high concentrations of NAD+ are required in order to see the putative conformational change, can this experiment be replicated in solution in the absence of DNA, that is in the absence of any PARylation?

Fig. 6: There is a lack of understanding for the biochemical basis for PARP ‘trapping’, and thus new approaches are worthy of performing and reporting. The authors find (as previously shown in reference #50 and in PMID 30088474), that there is no difference in the rate of release of PARP1 from DNA ± olaparib. They do, however, observe the same “change in conformation” at 1639 cm-1 as observed in Fig. 5 upon addition of NAD+. Additionally, they observe a loss of beta-sheet at 1620-1625 cm-1. It is confusing how they simultaneously observe a gain and a loss of beta-sheet.

This could be better explained.

Page 12: “Yet the exact mechanism….Our approach allowed a detailed analysis…” This reviewer is not convinced that novel insights relating PARylation to release of PARP1 from DNA have been provided by this work. Truly novel findings could be, for example, learning how many additions are needed, or if modification of specific amino acids are required. Also, finding that PARP1 modifies other biomolecules is not novel. Nonetheless, as mentioned above, the orthologous methods used in this manuscript, and their comparison to the existing literature, are a worthy contribution.

Page 13: Some of these discussion points are very interesting and positively contribute to the quality of the manuscript.

Reviewer #3 (Remarks to the Author):

Kruger et al. resolved details of the catalytic mechanism of post-translational modification by PARP1,. The authors developed and applied ATR FTIR spectroscopy as a means to trace the structural changes of the involved macromolecules and their complexes in a time-resolved manner. As a major result, the work shows that PARylation and PARP1 dissociation were most efficient at DNA nicks and 3’ phosphorylated ends. The allosteric role of NAD+ was demonstrated by developing an elegant isotope labeling approach. Moreover, the PARP inhibitors veliparib and olaparib exhibited the similar structural changes as binding of NAD+ to PARP1. This work represents an important step towards understanding of post-translational modifications in general by applying ATR FTIR as a label-free vibrational spectroscopic method.
Overall, the quality of the spectroscopic data is excellent and the drawn conclusions are sound. Thus, I recommend publishing the MS in your journal with minor revisions. As a spectroscopist, I will focus my queries on the experimental part and leave the discussion on the biological implications to my fellow expert reviewers.
The authors present real-time monitoring of PARylation. Yet, the affinity constants of the various complexes have not been given. If these are unknown, I am wondering if it is possible to determine the affinity constants by the applied ATR FTIR methodology?
Page 3, lines 7-8 “ATR-FTIR spectroscopy is a very sensitive method …”: As ATR FTIR spectroscopy is a well-known method in vibrational spectroscopy, the authors may insert references to review articles on the method (e.g. Goormaghtigh er al., BBA 1999, 1422, 105-185 and Nyquist et al. Chembiochem. 2004, 5, 431-436).
Page 4, line 11 “… which contained a multi-reflection silicon crystal with a light penetration depth of ~850 nm at 1000 cm-1.”: As the angle of incidence at the interface between the reflection element and the protein/water phase is varying over fairly large angles in a multi-reflection setup, the penetration depth may be better expressed in terms of a range (e.g. 850 nm +/- ?? nm) rather than a seemingly exact decay length.
Page 4, line 23 “Immobilization of biotinylated DNA strand break models”: The approach to tether DNA to the surface involved the well-known avidin/streptavidin interaction. Streptavidin is a fairly large protein which may undergo structural changes upon binding of the various substances. Such potential structural changes will be recorded more strongly as streptavidin is closer to the solid surface than the other components of the interaction complex, i.e. the evanescent electromagnetic field is stronger. The authors may comment on this.

Response to reviewers' comments for Ms NCOMMS-19-29775A:

We would like to thank all three referees for their thorough and thoughtful reviews and their helpful suggestions to further improve the manuscript. We included a considerable amount of new data and revised the manuscript according to the referees' requests and recommendations. Please find verbatim copies of the referees' comments and our replies below.

The major revisions are summarized in the following:

A: New experiments / incorporation of new data:

- A new control experiment was incorporated into the manuscript showing that in the absence of DNA no binding of PARP1 to streptavidin occurred (**new Suppl. Fig1C**).
- The PARylation status of PARP1 in the reaction supernatant was analyzed after the completion of the IR experiment (**new Suppl. Fig2E**). These data demonstrate that PARP1 in the reaction supernatant is highly auto-modified with poly(ADP-ribose).
- The gel-based PARylation assay shown in **original Suppl. Fig3D/E** was performed in higher replicate numbers and is now shown in **new Suppl. Fig3D**. To this end, the experimental protocol was slightly adapted to improve reproducibility and to make conditions more comparable to the ones used in IR experiments (i.e., change of buffer composition as reported in the Material and Methods part of the revised manuscript).
- The PARylation status of streptavidin was analysed in **new Fig4D**. The corresponding PARP1 control staining is shown in **new Suppl. Fig3E**. This important new data set proves that streptavidin is the major target for trans-PARylation at the surface of the ATR crystal.
- The PARylation status of DNA was analyzed in **new Suppl. Fig3F**. These data provide strong evidence that in our setting DNA is not a target for trans-PARylation.
- The PARP1 catalytic mutant PARP1-E988K, which exhibits mono-ADP-ribosylation activity, was analyzed using the ATR-FTIR approach. This new data set is presented in **new Suppl. Fig6**.
- Furthermore, potential changes in the secondary structure of PARP1 induced by NAD^+ were analyzed after preincubation with pharmacological PARP inhibitors. Data are presented in **new Suppl. Fig7A+B** and show that no changes occur in this case, which again demonstrates that structural changes are specifically induced by binding of NAD^+ or PARP inhibitors to PARP1's catalytic center of.

B: Revisions concerning data evaluation and data presentation:

- While preparing the revised manuscript, we noticed in the evaluation of data shown in **Fig5A** a wrong scaling in one out of three spectra. This was corrected in the revised manuscript. The conclusions drawn on that data did not change in any aspect.
- In all IR spectra, labelling of the x-axes was corrected from 'Wavelength' to 'Wavenumber'.

- In the 'IR difference spectra', labelling of the y-axes was corrected from 'Absorbance' to 'ΔAbsorbance'.
- To address an issue raised by reviewer #2, the presentation of the graph in **Fig2B** (i.e., PARP1 DNA binding kinetics) has been adapted. [*N.B.*: The data themselves are identical as compared to the original version of the manuscript.]
- To address an issue raised by reviewer #1, in **Fig2D** (i.e., comparison of PARP1 WT binding to different DNA molecules) the range of the y-axis has been adapted to the ones shown in figures 5 and 6 to allow better comparison of the different data sets.
- In figures **Fig3C+D** and **Fig4B+C** (i.e., showing the release of PARP1 from DNA and PAR formation kinetics after addition of NAD^+) the presentation of the graphs has been adapted. [N.B.: Data themselves are identical as compared to the original version of the manuscript.]
  → Since in the original version of the manuscript, the time point before addition of NAD^+ was included and set to '-1 min'; this data point was removed, as the exact time point is not known for technical reasons. This had no significant impact on kinetic values and did not change the validity of the data set or any conclusions drawn from this.
- A new figure has been incorporated as **Fig7** showing a schematic synopsis of the major findings of this study.
- Individual data points were included in bar graphs, as requested by 'Nature' guidelines.
- A new Excel table is included, listing all data points of the individual figures, as requested by 'Nature' guidelines.

<u>Specific responses:</u>

Color code:

Original text by reviewers: in black

Our reply: in blue

Quotations from the revised manuscript: in green

Reviewer #1 (Remarks to the Author):

"Krüger et al use the spectroscopic technique ATR-FTIR to report on the kinetics of PARP-1 (i) binding to DNA, (ii) catalyzing poly(ADP-ribose) formation, and (iii) releasing from DNA. They also investigate the influence of PARP inhibitors on this process. The study addresses interesting aspects of PARP-1 function, probably most importantly the complicated process of PARP-1 release from DNA due to poly(ADP-ribose) catalysis. While the focus of the study is interesting and the ATR-FTIR approach is novel, it is difficult to draw confident conclusions from the results.

Reply:

We thank the reviewer for acknowledging our work and for his/her valuable suggestions and recommendations. We are confident that the conclusions drawn in this study are robust and reliable, in particular after revising the manuscript according to the suggestions of all three referees.

The study lacks some controls that could test whether the interpretation of the results is correct."

Reply:

We have carried out several additional control experiments supporting our interpretations, which are included in the Supplementary Information section:

New **Suppl. Fig1C** demonstrates that PARP1 binds specifically to DNA strand break-mimicking oligonucleotides and that no binding to streptavidin occurs in the absence of DNA.

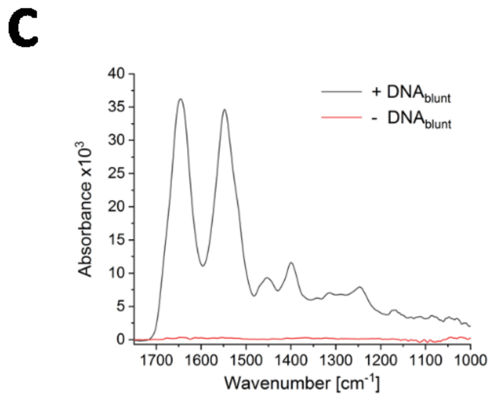

Suppl. Fig1. "(C) Comparison of PARP1 binding to DNA_{blunt}, which was immobilized via streptavidin ($+DNA_{blunt}$), or streptavidin alone, i.e., without DNA_{blunt} ($-DNA_{blunt}$)."

New **Suppl. Fig7A+B** show that no structural changes of PARP1 occurred upon addition of NAD^+ after preincubation with pharmacological PARP inhibitors. This result further supports that changes in the secondary structure of PARP1 are induced by binding of NAD^+ to the catalytic center.

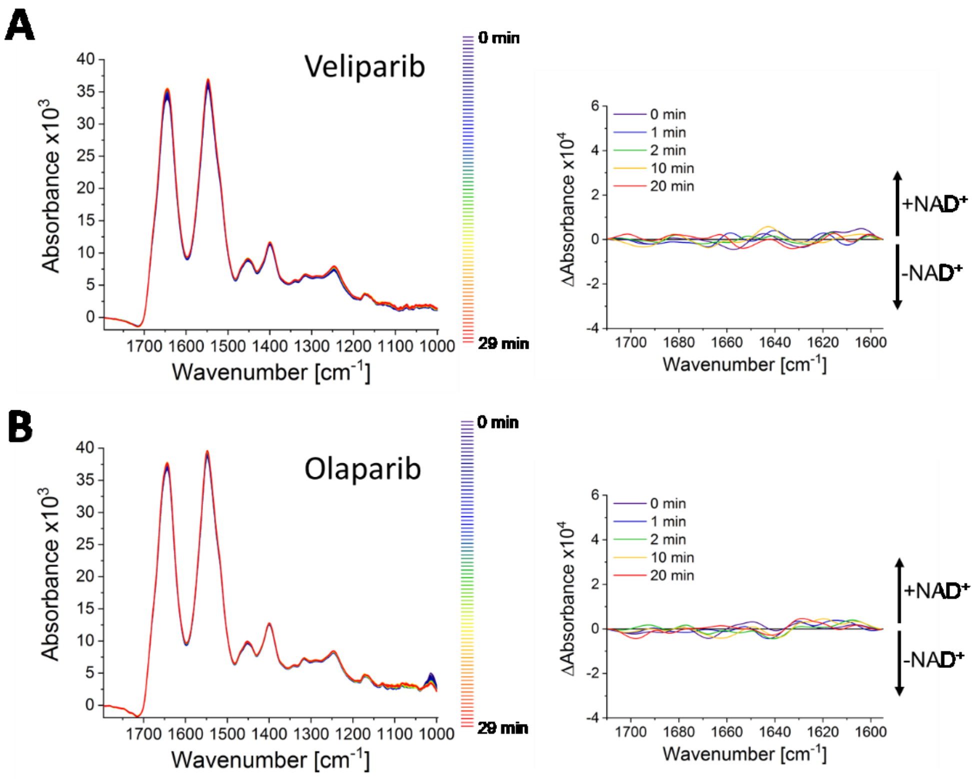

“**Suppl. Figure 7.** (A) Addition of 100 µM NAD^+ to PARP1 bound to immobilized DNA_{blunt} after preincubation with veliparib (A) or olaparib (B). *Left panels*: Time-dependent IR spectra. *Right panels*: Secondary structure analysis of PARP1. Difference spectra of amide I bands (1710-1595 cm^{-1}) before and after the addition of NAD^+ (0, 1, 2, 10 and 20 min) were calculated and do not indicate significant structural changes. These control experiments show that structural changes of PARP1 are specifically observed after binding of NAD^+ to the catalytic center.”

“Some of the difference spectra are stated as having non-significant features (Fig. 2D), but it is not clear why they are deemed to be non-significant.”

<u>Reply:</u>

In order to allow better comparison to data of other experiments, we adjusted the scale of the y-axis in **Fig2D** to the same range as shown in **Figs. 5 & 6**. This demonstrates that structural changes of PARP1 upon DNA binding are much weaker as compared to NAD^+ binding or to binding of pharmacological PARP inhibitors. In our view this justifies the expression ‘non-significant’. However, in order to avoid a potential misunderstanding of the expression ‘significant’ in statistical terms, we changed the wording to ‘substantial’. We thank the reviewer for this valuable comment.

D

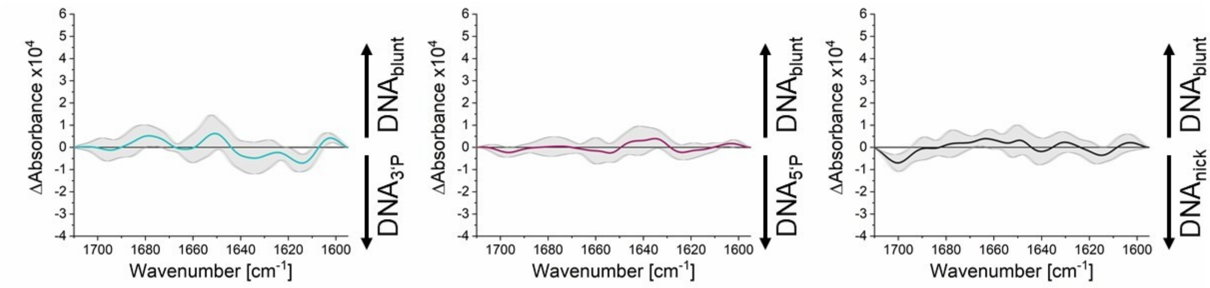

Fig2 "(D) Secondary structure analysis. Difference spectra of amide I bands of PARP1 bound to DNA_{blunt} and $DNA_{3'P}$, $DNA_{5'P}$ or DNA_{nick} were calculated. Average curves and SD (grey) of 9 difference spectra are plotted (3 independent experiments, respectively)."

"Some controls to demonstrate the power and the sensitivity of the technique are warranted. One way to address these issues would be to use mutant versions of PARP-1 that have known characteristics in terms of binding affinity or ability to be activated or ability to bind NAD+, and to demonstrate that these mutants introduce the expected changes in the ATR-FITR spectra."

Reply:

Apart from the new control experiments included in **Suppl. Fig1C** and **Suppl. Fig7A+B** (as mentioned above), we included a whole new data set on the PARP1 mutant $PARP1^{E988K}$ (new **Suppl. Fig6** and text on **p. 12/13**). The residue E988 is highly conserved among PARPs and it is part of the catalytic triad within the donor site (Alemasova and Lavrik 2019). It is one of the most widely studied PARP1 mutations, since mutations of this residue were shown to result in loss of PARylation activity, while maintaining MARylation activity (Marsischky, Wilson et al. 1995, Rolli, O'Farrell et al. 1997, Beneke, Scherr et al. 2010, Rank, Veith et al. 2016). In this new data set, we confirm the DNA binding ability and mono-ADP-ribosylation activity of the $PARP1^{E988K}$ mutant using the ATR-FTIR spectroscopic approach. We further show distinct structural differences between $PARP1^{E988K}$ and wild-type PARP1 (**Suppl. Fig6D**). Furthermore, we identified changes in difference spectra of the $PARP1^{E988K}$ +/- NAD^+ that were similar to those observed for wild-type PARP1, providing further evidence for the specificity of the structural changes observed for wild-type PARP1 upon NAD^+ binding.

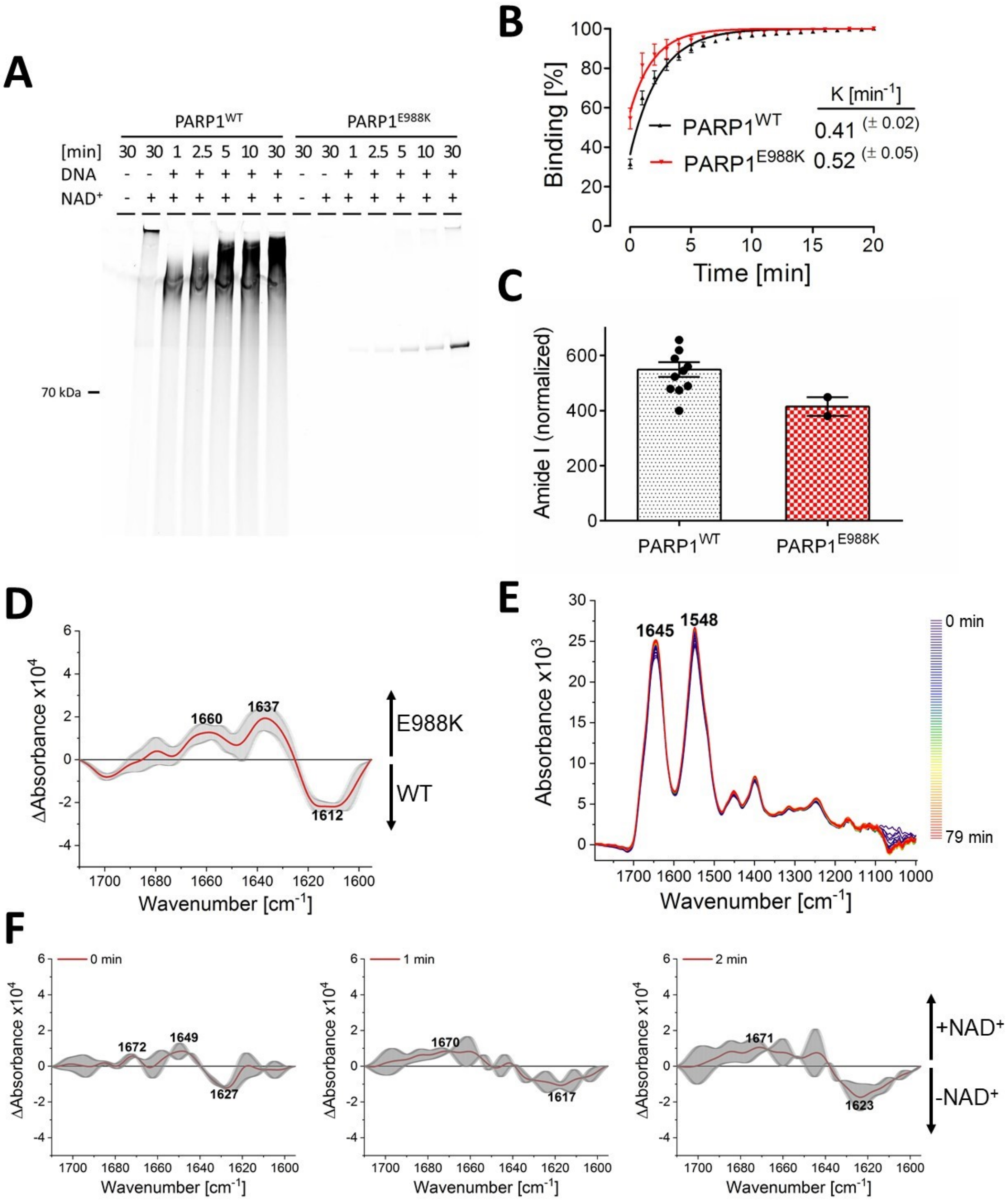

"Suppl. Figure 6. Analysis of PARP1 variant E988K. **(A)** Comparison of auto-modification of $PARP1^{WT}$ and $PARP1^{E988K}$ via a gel-based assay using fluorescently labelled NAD^+. PARylation was started by the addition of 100 µM TAMRA-labelled NAD^+. **(B)** Evaluation of time-dependent binding of $PARP1^{WT}$ and $PARP1^{E988K}$ to DNA_{blunt}. '0 min' refers to start of measurements. Signal intensities of amide I bands (1645 cm^{-1}) at 20 min were set to 100%. Binding kinetics were calculated via a mono-exponential fit function. $PARP1^{E988K}$: Means ± SEM of two independent experiments. Data of $PARP1^{WT}$ was taken from Figure 2. **(C)** Comparison of the amount of $PARP1^{WT}$ and $PARP1^{E988K}$ bound to DNA_{blunt} after 20 min of incubation. Amide I bands (1645 cm^{-1}) were normalized to the amount of immobilized DNA (1220 cm^{-1}). Data of $PARP1^{WT}$ was taken from Figure 2. **(D)** Secondary structure analysis. Difference spectra of amide I bands of $PARP1^{WT}$

and PARP1^{E988K} bound to DNA$_{blunt}$ were calculated. Average curves and SD (grey) of 6 difference spectra are plotted (PARP1^{WT}: 3 independent experiments; PARP1^{E988K}: 2 independent experiments). **(E)** Representative time-resolved spectra subsequent to the addition of 100 μM NAD$^+$ to PARP1^{E988K} bound to immobilized DNA$_{blunt}$. Amide I (1645 cm^{-1}) and amide II (1548 cm^{-1}) bands are indicated. **(F)** Secondary structure analysis of PARP1^{E988K} upon addition of 100 μM NAD$^+$. Difference spectra of amide I bands (1710-1595 cm^{-1}) before and after the addition of NAD$^+$ (0, 1 and 2 min) were calculated. Average curves and SDs (in grey) of 2 independent experiments are plotted respectively."

"As the study currently stands, it is not entirely clear that the observations have been interpreted correctly. This concern applies mainly to the interpretation of the ~1640 cm-1 band as a structural change in PARP-1."

Reply:

The experiments comparing the difference spectra using unlabeled and isotopically labeled NAD$^+$ give strong evidence for changes in the PARP1 secondary structure (**Fig5**). This is further substantiated by changes in difference spectra that have been observed after binding of pharmacological PARP inhibitors (**Fig6**) as well as in new experiments performed with the PARP1^{E988K} mutant (**Suppl. Fig6**). Additionally, we have performed experiments analyzing potential 'off-target' effects of NAD$^+$ binding by studying potential structural changes upon addition of NAD$^+$ to PARP1 that was preincubated with olaparib and veliparib (**Suppl. Fig7A+B**, see above). Taken together, these additional data provide a very robust foundation for the conclusions drawn. Nevertheless, we now discuss potential caveats in the discussion part of the revised manuscript, such as unexpected changes in the structure of streptavidin (**p. 16**).

"Another issue concerns the setup of the experiment. It appears that the majority of the poly(ADP-ribose) is attached to either the DNA or the streptavidin, so not the major target of PARP-1 modification. Thus, the build up of poly(ADP-ribose) on PARP-1 itself is not really being measured, and is instead inferred to exist. Would other attachment strategies (i.e. neutravidin, longer DNAs) potentially overcome this issue."

Reply:

We would like to thank the reviewer for this important comment. As the issue concering the nature of the trans-PARylation target at the crystal surface was raised by this reviewer (see below) and also by other reviewers, we addressed this point in detail in the revised manuscript. Our new experiments using gel-based assays clearly indicate that PARylation of streptavidin occurs (**Fig4D**), yet not of DNA (**Suppl. Fig3F**).

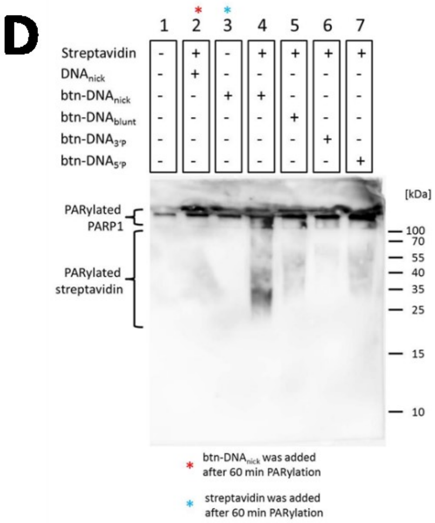

Fig4: "(D) Analysis of covalent PARylation of streptavidin in the presence of biotinylated (btn-DNA) and non-biotinylated DNA (DNA) via western blot and subsequent immunodetection of PAR. PARP1 (1 µM) and NAD^+ (500 µM) were present in all samples. Immunodetection of PARP1 is shown in Suppl. Figure 3E."

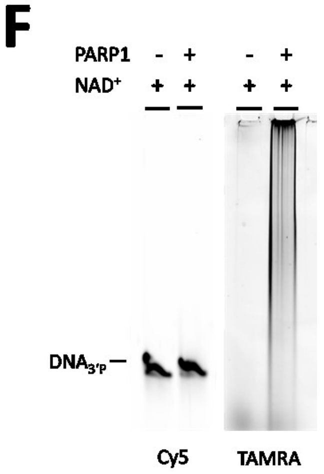

Suppl. Fig3: "(**F**) Analysis of covalent PARylation of $DNA_{3'P}$ via a gel-based assay using Cy5-labelled $DNA_{3'P}$ ('Cy5') and TAMRA-labelled NAD^+ ('TAMRA')."

Furthermore, in new **Suppl. Fig2**, we include control experiments analyzing the PARylation status of the PARP1 after its release from the crystal surface, which proves strong auto-modification of PARP1 in the supernatant after completion of the PARylation reaction. From these experiments it can be concluded that the large majority of PARylation in our system occurs at PARP1 itself. Yet, the trans-PARylation of streptavidin, which is immobilized at the crystal surface, is one of the big advantages of the present experimental system, since this serves as a very sensitive read-

out to analyze PARP1 activity. If PARylation of streptavidin did not occur, real-time monitoring of PAR formation would not be possible to the same extent, since auto-modified PARP1 would be presumably quickly released from the crystal surface and therefore undetectable. A similar mechanism of trans-PARylation may also apply in cells, when thinking of the presence of histones in a DNA/chromatin environment.

We took into account the reviewer's recommendation to test a longer DNA strand break-mimicking oligonucleotide. With a length of 60 bp, it is around double the size of the other oligonucleotides. The figure below shows that when using this longer DNA molecule, trans-PARylation of the crystal surface occurred to the same extent, as observed with the shorter DNA molecules, indicating that the spatial proximity provided by the longer DNA variant, is still sufficient to mediate the PARylation of streptavidin. (Please note that this data has not been included in the manuscript.) Since PARylation of streptavidin is very likely to be independent of its exact amino acid sequence, it is expected that testing of neutravidin would not make any significant difference regarding trans-PARylation of the crystal surface.

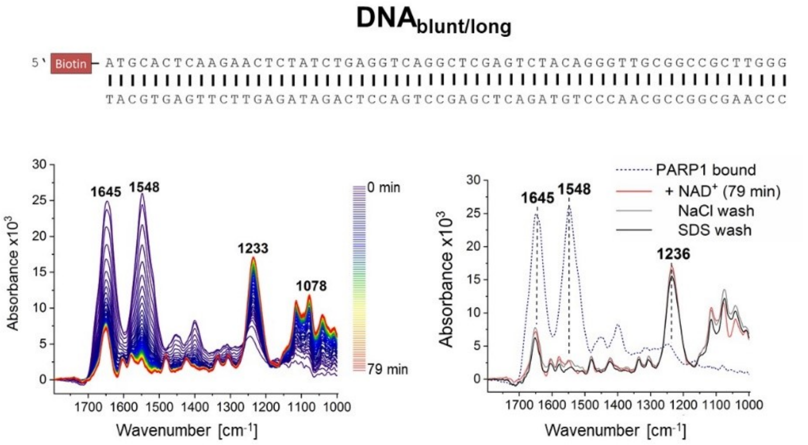

Figure legend: PARP1-dependent PARylation at immobilized $DNA_{blunt/long}$. *Lower left panel*: Time-dependent spectra subsequent to the addition of 100 µM NAD^+ to PARP1 bound to immobilized $DNA_{blunt/long}$. Amide I (1645 cm^{-1}) and amide II (1548 cm^{-1}) bands of PARP1 and anti-symmetric (1236 cm^{-1}) and symmetric (1074 cm^{-1}) phosphate vibrations of generated PAR are indicated. *Lower right panel*: Spectra of PARP1 bound to $DNA_{blunt/long}$ before ('PARP1 bound') and after the addition of 100 µM of NAD^+ ('79 min') and subsequent washing with 1 M NaCl ('NaCl wash') and 1% SDS ('SDS wash').

"Other concerns:

There are no error bars on Figure S3e, suggesting this experiment was only performed once. Since there are some conclusions drawn in comparing these data between the different DNAs used, the experiment should be done multiple times to provide SEM and a sense of the variability."

Reply:

The experiment has been repeated in three independent experiments and new data has been included in **Suppl. Fig3D**. Statistically significant differences in PARP1 activity were observed between DNA_{blunt} and $DNA_{5'P}$. Discussion of the data has been included on **p. 10**:

“Next, we compared our ATR-FTIR spectroscopic findings on the activation of PARP1 by different DNA strand break-mimicking oligonucleotides with an orthogonal approach using a conventional gel-based assay in combination with TAMRA-labelled NAD^+ (**Suppl. Figure 3D and E**). In accordance with the IR spectroscopic findings, PARylation of PARP1 activated by $DNA_{5'P}$ was significantly reduced. In contrast, we did not detect significant differences between PARylation of PARP1 activated by DNA_{blunt}, $DNA_{3'P}$ and DNA_{nick}, probably due to a lack of sensitivity of the gel-based assay. Those data demonstrate the high sensitivity and reproducibility of the ATR-FTIR spectroscopic approach in comparison to conventional biochemical assays...”

“The use of the term "allosteric binding" is confusing in some places: "...caused by allosteric binding of NAD+ to the catalytic centre.." "The allosteric binding of NAD+..."

It seems odd to refer to NAD+ binding as allosteric, unless talking specifically about the effect on DNA binding.”

Reply:

We thank the reviewer for this important comment. As pointed out by the reviewer, the term allosteric binding refers to the DNA binding ability of PARP1. We apologize for using this sometimes in an ambiguous manner. In the revised manuscript this has been clarified throughout the text. E.g., pp.1, 13, and 16.

“Can it be directly tested whether the DNA and/or the streptavidin are actually modified with poly(ADP-ribose)?”

Reply:

Please see response above.

“Figure 4: Legend says D, but there is no panel labelled as D.”

Reply:

Thanks for highly careful reading; this has been corrected.

“"Notably, as we observed strong automodification of PARP1 in the gel-based assay, this suggests that automodification of PARP1 mainly occurs, after initial activation, in the absence of DNA.”

Since the poly(ADP-ribose) produced in the ATR-FITR setup is not really directed at PARP-1, whereas it is directed toward PARP-1 in the gel-based assay, it does not seem safe to suggest the statement above.”

Reply:

Our new data included in **Suppl. Fig2E** (i.e., showing strong auto-modification of PARP1 after its release from the crystal surface) and **Fig4D** (i.e., showing that streptavidin serves an additional minor PARylation target) further support our original statement. While the reviewer is correct that we do not provide strong evidence to prove this hypothesis, we still think it is an interesting option that is worth discussing. To this end, we changed the wording of the sentence on **p. 15** into:

“Notably, as we detected strong auto-modification of PARP1 after its release from the crystal surface, but not or only to a minor extent at the crystal surface, this argues for the hypothesis that PARP1 auto-modification mainly occurs after its initial activation in the absence of DNA.”

Reviewer #2 (Remarks to the Author):

“The authors use ATR-FTIR to study the binding, activity (PARylation), and release of PARP1 from various forms of DNA damage. Overall the manuscript is very well written, and it is clear that the experiments were performed with great care and attention to detail. While the novelty of the findings presented in this manuscript is not overwhelming, confirmation of previous observations by this orthogonal and novel approach are a valuable contribution to the literature. I recommend acceptance upon minor modifications. See more detailed comments below:

We thank the reviewer for acknowledging our work and for his/her valuable suggestions and recommendations.

Fig. 2B: Should the percent binding not start at 0% in the fitting procedure? It appears to start at ~30%, and it seems that a fit through 0,0 would lead to non-first-order kinetics, which would be problematic for the interpretations made in the text.

Reply:

We thank the reviewer for this valuable comment. For technical reasons, there is a time gap of some seconds between the addition of PARP1 and the acquisition of the first data point. Thus, the time point ‘t = 0 min’ represents the first possible time point of measurement, however at this time point the binding reaction took place already for some seconds, therefore leading to starting values of ~30%. In order to clarify this issue and to avoid any misunderstanding we revised graphs, figure legends and the Material and Methods part accordingly.

“Additionally, under the conditions of 2 µM PARP1 and with an apparent rate of ~0.5/min, one can calculate on-rates of ~4 x 103 M-1s-1. These values are very slow compared to the rates of association of most DNA-binding proteins (>>106 M-1s-1), in particular the measured value for PARP1 of 109 M-1s-1 (see PMID 30088474). Does the apparent slow on-rate have something to do with the surface chemistry of the experimental system? The authors need to address these issues of data-fitting, interpretation, and comparison to existing literature.”

Reply:

We thank the reviewer for addressing this important issue, which is clarified as follows: Calculations of absolute values of binding constants and comparison with the values, e.g., as reported in reference PMID 30088474, are not applicable with our approach, because the exact amount of bound PARP1 is unknown, which limits the determination of absolute values for binding constants. The reason for this is that we need to apply DNA/streptavidin in excess to obtain saturating binding conditions at the crystal surface. As evident from the **data shown below**, only a minor portion of the total amount of DNA is actually immobilized at the crystal surface, and therefore, in turn, only a minor portion of PARP1 is bound to immobilized DNA (see **Suppl. Fig2E**, compare the loss of PARP1 signal between lanes 1 and 2).

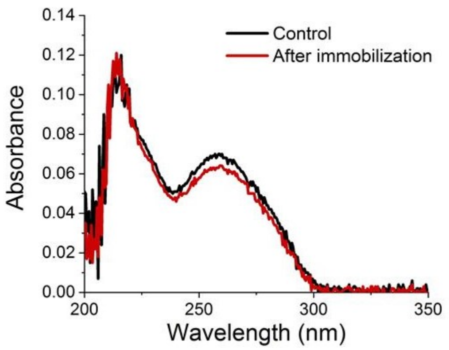

Figure legend: Determination of the immobilization efficiency of biotinylated DNA_{blunt} at the modified ATR crystal surface via streptavidin. UV/Vis-spectrum of 15 pmol DNA_{blunt} mixed with 10 pmol streptavidin was acquired before (‘Control’) and after 50 min incubation on the biotinylated crystal surface (‘After immobilization’). This shows that only a minor portion of the total amount of DNA is immobilized at the crystal surface. The data is shown for review purposes only and not included into the manuscript.

However, the curve fitting constants determined from our kinetic data as provided in the manuscript are highly suitable to compare the binding kinetics of PARP1 to different DNA molecules in relative terms. We included a clarifying statement on this issue in the Material and Methods part on **p. 5**:

"It is important to note that the experimental approach limits the calculation of absolute values of rate constants as the exact amount of immobilized DNA is an unknown parameter. Furthermore, due to the fast binding of PARP1 to the immobilized DNA, a time gap of some seconds between addition of PARP1 and the start of data acquisition cannot be avoided for technical reasons. Nevertheless, the method is highly suitable for a relative comparison of binding kinetics."

Furthermore, we now report on-rates as reference values previously determined in PMID 30088474 on **p. 2** of the revised manuscript:

"Fast and efficient cellular DNA repair is essential to ensure genomic integrity over time. One of the first signalling events upon DNA damage is the recruitment of poly(ADP-ribose) polymerase 1 (PARP1) to sites of DNA damage, in particular DNA strand breaks. This is a very fast process, where association rates of 10^9 $M^{-1}s^{-1}$ have been reported (Rudolph, Mahadevan et al. 2018)."

„Fig. 3B,C: The authors do a nice job of discriminating PAR formation and dissociation of PARP1, and their washing experiments nicely demonstrate that PARylation of non-self (DNA and/or streptavidin) must be occurring. As no one has previously demonstrated PARylation of streptavidin, a simple TAMRA-labeled NAD+ - based assay using soluble PARP1 and soluble streptavidin would be a good supplementary figure to include here."

Reply:

We thank the reviewer for this valuable comment appreciating the power and sensitivity of our spectroscopic approach. The issue of the nature of the trans-PARylation target was also raised by other reviewers (see above), we addressed this point in detail in the revised manuscript. We performed new experiments using gel-based assays which clearly indicate the PARylation of streptavidin (new **Fig4D**), yet not of DNA (new **Suppl. Fig3F**). In new **Suppl. Fig2**, we include further control experiments analyzing the PARylation status of the PARP1 molecules released from the crystal surface in the spectroscopic ATR-FTIR experiments. These controls proved strong auto-modification of PARP1 in the supernatant after completion of the PARylation reaction. From those experiments, it can be concluded that the large majority of PARylation in our system occurs at PARP1 molecules themselves. However, the trans-PARylation of streptavidin, which is immobilized at the crystal surface, provides a big advantage of this system, since this serves as a very sensitive read-out to analyze PARP1 activity in real-time by monitoring IR absorbance. A similar mechanism of trans-PARylation may also apply in cells, in consideration of the fact that histones are present in a DNA/chromatin environment.

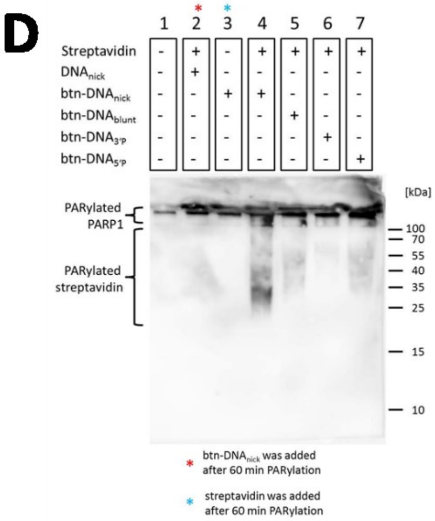

Fig4: “(**D**) Analysis of covalent PARylation of streptavidin in the presence of biotinylated (btn-DNA) and non-biotinylated DNA (DNA) via western blot and subsequent immunodetection of PAR. PARP1 (1 μM) and NAD^+ (500 μM) were present in all samples. Immunodetection of PARP1 is shown in Suppl. Figure 3E.”

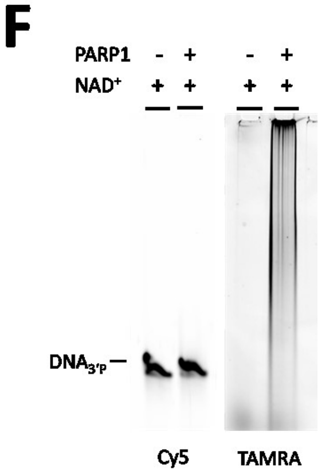

Suppl. Fig3: “(**F**) Analysis of covalent PARylation of $DNA_{3'P}$ via a gel-based assay using Cy5-labelled $DNA_{3'P}$ (‘Cy5’) and TAMRA-labelled NAD^+ (‘TAMRA’).”

Fig. 3D: My back-of-the-envelope calculations (assuming 10 pmol of DNA, each bound to 1 PARP1 with a turnover number of ~5 s-1) suggests that the amount of NAD+ consumption is about as expected from solution-based measurements, which is reassuring and suggests that PARP1 bound to the surface is intact and catalytically active. A comparison to the existing literature re: turnover numbers could be made here and would strengthen the authors’ conclusions.

Reply:

Since enzymatic parameters of PARP1 have been reported already previously (Altmeyer, Messner et al. 2009), we focused in this study on the analyses of PARP activity in relative term. Furthermore, our approach does not allow to calculate turnover rates in absolute terms, as the exact amounts of immobilized DNA and PARP1 are unknown (see above). However, both the IR data (**Fig3**) and the analysis of auto-modified PARP1 that has been released from the crystal surface (new **Suppl. Fig2E**) clearly demonstrate that the rec. PARP1 used in the experiments is intact and catalytically active. Furthermore, the kinetic parameters that are presented in the manuscript allow relative comparison of PARP1 activity and release from DNA between different conditions in a time-resolved manner (i.e., at different NAD^+ concentrations and upon binding to different DNA strand break-mimicking oligonucleotides).

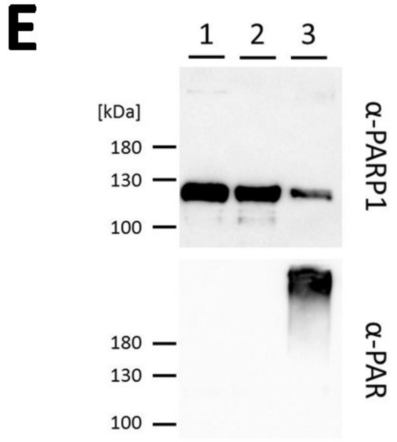

Suppl. Fig. 2: "(E) Analysis of the supernatant from an ATR-FTIR spectroscopic experiment by immunoblotting. PARP1 was detected via CII10 and PAR was detected via 10H antibody. 1+2: Analysis of the efficiency of PARP1 binding to immobilized DNA_{blunt} [before (1) and after (2) addition of PARP1 to immobilized DNA_{blunt}]. 3: Analysis of the extent of auto-modification of PARP1 after NAD^+ addition (79 min)."

"Fig. 4: Although the differences in rates of PARylation and PARP1 release are relatively minor, the correlation between automodification and release is as expected, thus making these data believable. Given that the differences between the different forms of DNA damage are less than 2-fold, the interpretations presented in the Discussion appear too strong."

Reply:

We changed the wording in the 'Discussion' on **p. 14** according to the recommendation of the referee:

"Nevertheless, the fact that PARP1 was in principle active at all tested DNA strand break structures and that differences in PARylation activities between the different DNA strand break

mimicking oligonucelotides were less than 2-fold is in accordance with an assumed redundancy between PARP1 and PARP2 (Menissier de Murcia, Ricoul et al. 2003). While each of them might have preferential DNA structures for activation, and thereby fulfil unique roles in cellular processes, losing one of them can at least partly be compensated by the other. It is worth mentioning that recent data analysing the activation of PARP1 at various types of DNA strand breaks via a colorimetric assay did not result in significant differences in PAR formation (Langelier, Riccio et al. 2014). Still, there was a trend of preferred activation of PARP1 at 3' phosphorylated ends, which demonstrates the high sensitivity and reproducibility of the IR spectroscopic approach in comparison to conventional methods used to study PARylation."

"Fig. 5: The care with which the authors perform the analyses of changes in the conformation of PARP1 upon PARylation are commendable. That is, using the "trick" of isotopically labeled NAD+ in order to better resolve the signal of interest is excellent. Also, it is evident that they have carefully considered concentrations of NAD+ and extents of reaction in their sample preparation. The data are convincing but shed little insight into the conformational changes that occur upon binding of NAD+. The finding of a slight increase in some disordered protein or more beta-sheets, although correlating with Pascal's allosteric-mechanism (reference #26), does not actually identify which part of the protein is undergoing this conformational change. Given that high concentrations of NAD+ are required in order to see the putative conformational change, can this experiment be replicated in solution in the absence of DNA, that is in the absence of any PARylation?"

Reply:

We agree with the reviewer that IR spectroscopy in general is limited to the analysis of protein secondary structures and does not allow single-amino-acid resolution. Still, with this study we provide first evidence that distinct changes in PARP1 secondary structure occur upon NAD^+ and PARP inhibitor binding. It is important to note that the strength of this study is given by the label-free and time-resolved approach to analyze several steps of DNA-damage-dependent and PARP1-mediated PARylation in one setup with high chemical specificity and temporal resolution – and above that, to some extent also structural resolution. The suggestion of the reviewer to replicate the experiment in solution in the absence of DNA is interesting, however, for technical reasons not applicable in our setting, since the binding of PARP1 to DNA is crucial in order to immobilize PARP1 at the crystal surface. Without PARP1 bound to immobilized DNA no signal can be obtained in IR measurements. In order to further strengthen data and conclusions on changes in PARP1's secondary structure upon NAD^+ binding, we incorporated new control experiments. Thus, when adding NAD^+ to PARP1 that had been preincubated with pharmacological inhibitors, this did not lead to any additional structural changes of PARP1, indicating that the observed changes are indeed caused by binding to the catalytic center (**Suppl. Fig7**). Furthermore, we included a new data set in **Suppl. Fig6**, in which we analyzed the mutant $PARP1^{E988K}$, which exhibits mono-ADP-ribosyl transferase activity only. While observing notable structural differences between wild-type PARP1 and the $PARP1^{E988K}$ mutant, we also observed specific structural changes in the $PARP1^{E988K}$ mutant upon NAD^+ binding at a wavenumber of

1649 cm^{-1}. This again indicates that binding of NAD^+ to PARP1's catalytic center induces specific changes in the secondary structure of PARP1.

"Fig. 6: There is a lack of understanding for the biochemical basis for PARP 'trapping', and thus new approaches are worthy of performing and reporting. The authors find (as previously shown in reference #50 and in PMID 30088474), that there is no difference in the rate of release of PARP1 from DNA ± olaparib. They do, however, observe the same "change in conformation" at 1639 cm-1 as observed in Fig. 5 upon addition of NAD+. Additionally, they observe a loss of beta-sheet at 1620-1625 cm-1. It is confusing how they simultaneously observe a gain and a loss of beta-sheet. This could be better explained."

Reply:

We thank the reviewer for this valuable comment and modified the text accordingly on **p.13** in order to clarify this issue.

"Interestingly, veliparib as well as olaparib resulted in a positive band at ~1639 cm^{-1}. The position of the band was similar to the one observed after addition of NAD^+ (**Figure 5A and D**) and can be assigned to disordered or β-sheet structures (Barth 2007). This finding points to a common binding mechanism with similar structural consequences for PARP1. Moreover, a negative band was observed in the region 1620-1625 cm^{-1}, which can be assigned to β-sheet structures as well (Barth 2007). In contrast to the position at ~1639 cm^{-1}, the position at 1620-1625 cm^{-1} is more representative for extended β-sheet structures with stronger hydrogen bonds (Chirgadze and Nevskaya 1976, Krimm and Bandekar 1986, Barth 2007). This suggests a loss of this type of β-sheet structures upon binding to veliparib or olaparib."

Furthermore, we included PMID 30088474 as a reference on p. 14 and p.17 in the revised version of the manuscript.

"Page 12: "Yet the exact mechanism….Our approach allowed a detailed analysis…" This reviewer is not convinced that novel insights relating PARylation to release of PARP1 from DNA have been provided by this work. Truly novel findings could be, for example, learning how many additions are needed, or if modification of specific amino acids are required. Also, finding that PARP1 modifies other biomolecules is not novel. Nonetheless, as mentioned above, the orthologous methods used in this manuscript, and their comparison to the existing literature, are a worthy contribution."

Reply:

We would like to stress that the uniqueness, novelty and major impact of this study lays in the holistic, label-free, and time-resolved approach, which allows to study several important biochemical and biophysical aspects of PARP1-dependent PARylation with high analytical

sensitivity, chemical specificity, as well as temporal and to some extent also structural resolution. To the best of our knowledge, no other approach has so far covered DNA strand break-induced PARP1-dependent PARylation in such a comprehensive manner in one single experimental system. In our opinion and as pointed out by referee #3, this, in addition, represents an important step towards the understanding of post-translational modifications in general by applying ATR-FTIR spectroscopy as a label-free method. We put more emphasis on these aspects in the revised manuscript (e.g., **p.17**):

In conclusion, this study provides detailed insights into the dynamic and overlapping processes of PARP1-dependent PARylation including activation at DNA strand breaks, NAD^+ binding, PARylation and dissociation of PARP1 from DNA. The direct and label-free spectroscopic tracking of the enzymatic reaction enabled high sensitivity and reproducibility and revealed information on kinetics as well as on the tightly controlled structural changes of PARP1. Since PARP1 is a promising target for precision therapy by selectively treating DNA repair-deficient cancers, the detailed examination of the underlying molecular processes of PARP1-dependent PARylation can give us a starting point to improve our methodologies and develop future therapies. Finally, the ATR-FTIR spectroscopic approach not only provides an ideal platform to study molecular processes of PARP1-dependent PARylation, but has also the potential to unravel the enzymatic mechanisms of other enzymes involved in DNA metabolism as well as to provide a deeper understanding of how post-translational modifications in general control enzymatic functions and structures.

“Page 13: Some of these discussion points are very interesting and positively contribute to the quality of the manuscript.”

<u>Reply:</u>

We thank the reviewer for acknowledging the quality of our work.

Reviewer #3 (Remarks to the Author):

“Kruger et al. resolved details of the catalytic mechanism of post-translational modification by PARP1,. The authors developed and applied ATR FTIR spectroscopy as a means to trace the structural changes of the involved macromolecules and their complexes in a time-resolved manner. As a major result, the work shows that PARylation and PARP1 dissociation were most efficient at DNA nicks and 3’ phosphorylated ends. The allosteric role of NAD+ was demonstrated by developing an elegant isotope labeling approach. Moreover, the PARP inhibitors veliparib and olaparib exhibited the similar structural changes as binding of NAD+ to PARP1. This work

represents an important step towards understanding of post-translational modifications in general by applying ATR FTIR as a label-free vibrational spectroscopic method.

Overall, the quality of the spectroscopic data is excellent and the drawn conclusions are sound. Thus, I recommend publishing the MS in your journal with minor revisions. As a spectroscopist, I will focus my queries on the experimental part and leave the discussion on the biological implications to my fellow expert reviewers.

We thank the reviewer for acknowledging our work and for his/her valuable suggestions and recommendations.

The authors present real-time monitoring of PARylation. Yet, the affinity constants of the various complexes have not been given. If these are unknown, I am wondering if it is possible to determine the affinity constants by the applied ATR FTIR methodology?"

Reply:

As reported by (Langelier, Riccio et al. 2014) the affinity constants for blunt-ended DNA and 5'-P DNA have been determined to be 7.6 nM and 6.2 nM, respectively. This information has been added in the revised manuscript on **p. 7**.

In principle it is possible to determine affinity constants by the ATR-FTIR methodology. However, a quite high amount of protein is required for a quantitative analysis. Because of limiting amounts of available recombinant protein, this was not in the focus of the current study.

"Page 3, lines 7-8 "ATR-FTIR spectroscopy is a very sensitive method …": As ATR FTIR spectroscopy is a well-known method in vibrational spectroscopy, the authors may insert references to review articles on the method (e.g. Goormaghtigh er al., BBA 1999, 1422, 105-185 and Nyquist et al. Chembiochem. 2004, 5, 431-436)."

Reply:

We included the references as suggested.

"Page 4, line 11 "… which contained a multi-reflection silicon crystal with a light penetration depth of ~850 nm at 1000 cm-1.": As the angle of incidence at the interface between the reflection element and the protein/water phase is varying over fairly large angles in a multi-reflection setup, the penetration depth may be better expressed in terms of a range (e.g. 850 nm +/- ?? nm) rather than a seemingly exact decay length."

Reply:

The reviewer is correct, that the penetration depth is not an exact value, but depends on the angle of incidence which varies at the interface between the internal reflection element and the protein sample, and this displacement occurs at each reflection point, thus summing up in a multi-reflection setup as we have used. In addition, the penetration depth depends on the wavenumber and the assumed refractive index of the protein sample with n ~ 1.5. This is the reason why we have written "~ 850 nm". To clarified on **p. 4** that this is only an approximate number:

"Real-time ATR-FTIR spectroscopic measurements were performed as described previously (Krüger, Bürkle et al. 2018, Kruger, Stier et al. 2019), with some adaptations. A Vertex 70V spectrometer (Bruker) was equipped with a BioATR cell II (Bruker), which contained a multi-reflection silicon crystal. The penetration depth of the IR beam into the sample depends on the wavenumber, refractive indices, and angle of incidence and is about 850 nm (calculated for 1000 cm^{-1}, n_{sample} = 1.5, $n_{silicon}$ = 3.4, and 45° angle of incidence. The spectral resolution was set to 4 cm^{-1}, and for each spectrum 100 scans were performed. The temperature of the crystal was controlled via an external water bath and set to 20°C. Unless otherwise stated, measurements were performed in Tris buffer (50 mM Tris pH 7.4, 150 mM NaCl)."

"Page 4, line 23 "Immobilization of biotinylated DNA strand break models": The approach to tether DNA to the surface involved the well-known avidin/streptavidin interaction. Streptavidin is a fairly large protein which may undergo structural changes upon binding of the various substances. Such potential structural changes will be recorded more strongly as streptavidin is closer to the solid surface than the other components of the interaction complex, i.e. the evanescent electromagnetic field is stronger. The authors may comment on this."

Reply:

A discussion of this issue has been added in the revised version of the manuscript on **p. 16**:

In our ATR-FTIR spectroscopic approach, DNA strand break models were immobilized via streptavidin at the crystal surface. In principle, it should be considered that streptavidin could undergo structural changes during measurements as well, which could not be distinguished from structural changes of PARP1. However, such structural changes are rather unlikely, since it is known that streptavidin is an extremely stable protein (Kurzban, Bayer et al. 1991, Alvarez-Gonzalez, Spring et al. 1999) and we could show previously that even elevated temperatures or high concentrations of detergents did not result in significant structural changes of streptavidin (Krüger, Bürkle et al. 2018). Furthermore, we can exclude that PARylation of streptavidin induced the structural changes at the band position ~1640 cm^{-1}, since changes at this band position were also observed upon application of PARP inhibitors instead of NAD^+. Finally, the controls included in this study, e.g. analysis of potential NAD^+-induced effects in the presence of PARP inhibitor or analysis of the PARP1 variant $PARP1^{E988K}$, provide strong evidence for PARP1-specific effects.

References

Alemasova, E. E. and O. I. Lavrik (2019). "Poly(ADP-ribosyl)ation by PARP1: reaction mechanism and regulatory proteins." Nucleic Acids Res.
Altmeyer, M., S. Messner, P. O. Hassa, M. Fey and M. O. Hottiger (2009). "Molecular mechanism of poly(ADP-ribosyl)ation by PARP1 and identification of lysine residues as ADP-ribose acceptor sites." Nucleic Acids Res **37**(11): 3723-3738.
Alvarez-Gonzalez, R., H. Spring, M. Muller and A. Burkle (1999). "Selective loss of poly(ADP-ribose) and the 85-kDa fragment of poly(ADP-ribose) polymerase in nucleoli during alkylation-induced apoptosis of HeLa cells." J Biol Chem **274**(45): 32122-32126.
Barth, A. (2007). "Infrared spectroscopy of proteins." Biochimica et Biophysica Acta (BBA) - Bioenergetics **1767**(9): 1073-1101.
Beneke, S., A. L. Scherr, V. Ponath, O. Popp and A. Bürkle (2010). "Enzyme characteristics of recombinant poly(ADP-ribose) polymerases-1 of rat and human origin mirror the correlation between cellular poly(ADP-ribosyl)ation capacity and species-specific life span." Mech Ageing Dev **131**(5): 366-369.
Chirgadze, Y. N. and N. A. Nevskaya (1976). "Infrared spectra and resonance interaction of amide-I vibration of the antiparallel-chain pleated sheet." Biopolymers **15**(4): 607-625.
Krimm, S. and J. Bandekar (1986). "Vibrational spectroscopy and conformation of peptides, polypeptides, and proteins." Adv Protein Chem **38**: 181-364.
Krüger, A., A. Bürkle, A. Mangerich and K. Hauser (2018). "A combined approach of surface passivation and specific immobilization to study biomolecules by ATR-FTIR spectroscopy." Biomedical Spectroscopy and Imaging **7**: 25-33.
Kruger, A., A. Stier, A. Fischbach, A. Burkle, K. Hauser and A. Mangerich (2019). "Interactions of p53 with poly(ADP-ribose) and DNA induce distinct changes in protein structure as revealed by ATR-FTIR spectroscopy." Nucleic Acids Res **47**(9): 4843-4858.
Kurzban, G. P., E. A. Bayer, M. Wilchek and P. M. Horowitz (1991). "The quaternary structure of streptavidin in urea." J Biol Chem **266**(22): 14470-14477.
Langelier, M.-F., A. A. Riccio and J. M. Pascal (2014). "PARP-2 and PARP-3 are selectively activated by 5′ phosphorylated DNA breaks through an allosteric regulatory mechanism shared with PARP-1." Nucleic Acids Research **42**(12): 7762-7775.
Langelier, M. F., A. A. Riccio and J. M. Pascal (2014). "PARP-2 and PARP-3 are selectively activated by 5' phosphorylated DNA breaks through an allosteric regulatory mechanism shared with PARP-1." Nucleic Acids Res **42**(12): 7762-7775.
Marsischky, G. T., B. A. Wilson and R. J. Collier (1995). "Role of glutamic acid 988 of human poly-ADP-ribose polymerase in polymer formation. Evidence for active site similarities to the ADP-ribosylating toxins." J Biol Chem **270**(7): 3247-3254.
Menissier de Murcia, J., M. Ricoul, L. Tartier, C. Niedergang, A. Huber, F. Dantzer, V. Schreiber, J. C. Ame, A. Dierich, M. LeMeur, L. Sabatier, P. Chambon and G. de Murcia (2003). "Functional interaction between PARP-1 and PARP-2 in chromosome stability and embryonic development in mouse." Embo j **22**(9): 2255-2263.
Rank, L., S. Veith, E. C. Gwosch, J. Demgenski, M. Ganz, M. C. Jongmans, C. Vogel, A. Fischbach, S. Buerger, J. M. Fischer, T. Zubel, A. Stier, C. Renner, M. Schmalz, S. Beneke, M. Groettrup, R. P. Kuiper, A. Burkle, E. Ferrando-May and A. Mangerich (2016). "Analyzing structure-function relationships of

artificial and cancer-associated PARP1 variants by reconstituting TALEN-generated HeLa PARP1 knock-out cells." Nucleic Acids Res **44**(21): 10386-10405.
Rolli, V., M. O'Farrell, J. Menissier-de Murcia and G. de Murcia (1997). "Random mutagenesis of the poly(ADP-ribose) polymerase catalytic domain reveals amino acids involved in polymer branching." Biochemistry **36**(40): 12147-12154.
Rudolph, J., J. Mahadevan, P. Dyer and K. Luger (2018). "Poly(ADP-ribose) polymerase 1 searches DNA via a 'Monkey Bar' mechanism." eLife **7**: e37818.

REVIEWERS' COMMENTS:

Reviewer #1 (Remarks to the Author):

The authors have made a nice effort to address the reviewer comments. The addition of the E988K mutant helps the presentation of the work.

I think the paper represents an interesting approach to studying PARP1 function. There are a few places where I feel that the authors have gone a bit too far in advertising the power of the technique. For example:

"we provide detailed information on the molecular consequences during distinct steps of the PARylation reaction"

"we have unravelled the molecular dynamics..."

It is not exactly clear what is meant by these statements, but to me and potentially to other readers, it implies a level of detail that the described technique does not have the power to provide. Even if all structural information of a protein is encoded in the collected data, there is no method to assign this information to specific parts of a protein molecule. Thus, I feel the comments above and others like it should be carefully worded to reflect the realities of the approach.

Reviewer #2 (Remarks to the Author):

This manuscript has improved significantly as a result of the review process and should be published. The authors are to be commended for their careful and thorough response.

My only remaining confusion is with regard to the kinetic assays in line 353ff, and then again in the Discussion line 530ff:

PARP bound to 5'P is the fastest at PARylation AND at falling off, yet the least total amount of it falls off. Can the authors provide some kind of interpretation that makes sense? (I believe the results, I just don't know how to think about them.)
thanks

Reviewer #3 (Remarks to the Author):

the authors have carefully revised their manuscript according to my suggestions. The manuscript is acceptable for publication.

General response:

We would like to thank all three referees again for their valuable comments improving our manuscript and their recommendation to publish our manuscript in Nature Communications. We addressed remaining issues below.

Specific responses to reviewers' comments:

REVIEWERS' COMMENTS:

Reviewer #1 (Remarks to the Author):

The authors have made a nice effort to address the reviewer comments. The addition of the E988K mutant helps the presentation of the work.

I think the paper represents an interesting approach to studying PARP1 function. There are a few places where I feel that the authors have gone a bit too far in advertising the power of the technique. For example:

"we provide detailed information on the molecular consequences during distinct steps of the PARylation reaction"

"we have unravelled the molecular dynamics..."

It is not exactly clear what is meant by these statements, but to me and potentially to other readers, it implies a level of detail that the described technique does not have the power to provide. Even if all structural information of a protein is encoded in the collected data, there is no method to assign this information to specific parts of a protein molecule. Thus, I feel the comments above and others like it should be carefully worded to reflect the realities of the approach.

Response:

We thank the reviewer for appreciating our experimental and textual revisions. We understand his/her concerns and carefully reworded our statements on p.1, p. 10, p. 26.

Reviewer #2 (Remarks to the Author):

This manuscript has improved significantly as a result of the review process and should be published. The authors are to be commended for their careful and thorough response.

My only remaining confusion is with regard to the kinetic assays in line 353ff, and then again in the Discussion line 530ff:

PARP bound to 5'P is the fastest at PARylation AND at falling off, yet the least total amount of it falls off. Can the authors provide some kind of interpretation that makes sense? (I believe the results, I just don't know how to think about them.) thanks

Response:

We thank the reviewer for acknowledging the revisions we have performed.

The reviewer raises an interesting point here. Although the data he/she is referring to (Fig. 4) indicate some trends, the differences in kinetics are small. Regarding the SEMs (Fig. 4b, c), the differences between the kinetics are not solid enough to draw conclusions for a meaningful interpretation. Thanks to the reviewer's comment, we realized that our text phrasing (previously in line 353ff, p. 10 – after formatting revision now on p.6, and in line 530ff, p. 14 – now on p. 10) might be overinterpreted and clarified these text passages accordingly. At this point, the relevance of the fast kinetics of PARP1 dissociation from 5' phosphorylated DNA ends and PAR formation is not clear yet. This would be an interesting follow-up study.

Reviewer #3 (Remarks to the Author):

The authors have carefully revised their manuscript according to my suggestions. The manuscript is acceptable for publication.

Response:

We thank the reviewer again for his/her valuable comments that helped us to improve the manuscript.
